# The association between maternal psychological stress and inflammatory cytokines in allergic young children

**DOI:** 10.7717/peerj.1585

**Published:** 2016-01-18

**Authors:** Mayumi Tsuji, Chihaya Koriyama, Megumi Yamamoto, Ayumi Anan, Eiji Shibata, Toshihiro Kawamoto

**Affiliations:** 1 Department of Environmental Health, University of Occupational and Environmental Health, Kitakyusyu, Japan; 2 Department of Environmental Toxicology, University of California, Davis, United States; 3 Department of Epidemiology and Preventive Medicine, Kagoshima University, Kagoshima, Japan; 4 Integrated Physiology Section, Department of Basic Medical Science, National Institute for Minamata Disease, Minamata, Japan; 5 Department of Clinical Nursing, School of Health Sciences, University of Occupational and Environmental Health, Kitakyusyu, Japan; 6 Department of Obstetrics and Gynecology, University of Occupational and Environmental Health, Kitakyusyu, Japan

**Keywords:** Allergy, Psychological stress, Children, Cytokines

## Abstract

**Background.** Previous studies have shown that psychological stress is linked to asthma prevalence. Parental psychological stress may potentially influence inflammatory responses in their allergic children. The purpose of this study is to clarify the association between maternal psychological status and inflammatory response of allergic young children.

**Methods.** The study subjects were 152 young allergic children (median age: 13 months) who had not shown any allergic symptoms in the past one month. mRNA expression levels of the inflammatory response genes IL-6, IL-8, IL-10 and IL-22 were quantified by qRT-PCR. Maternal psychological status was assessed by standardized questionnaires: the Centre for Epidemiological Studies Depression Scale (CES-D) for depression and the Japanese Perceived Stress Scale (JPSS) for perceived stress.

**Results.** A significant positive association was observed between maternal CES-D scores and IL-6 mRNA expression in the children with asthma. The JPSS scores were also positively associated with IL-8 mRNA expression in asthmatic children and IL-6 mRNA expression in children with allergic rhinitis. Similar trends were observed among children positive for house dust mite-specific IgE, but these associations were not significant.

**Conclusion.** This study supports the hypothesis that maternal psychological stress affects the inflammatory response in their allergic children.

## Introduction

The incidence of childhood allergic disease has increased recently throughout the world, particularly in westernized countries. Psychological stress, if sustained, can adversely affect critical functions, such as immune surveillance (Glaser, 2005), gastrointestinal integrity (Meddings & Swain, 2000) and compromise host defenses against viral infections (Steelman et al., 2009). Previous studies have documented a relationship between psychological stress and allergic diseases at all ages. Sandberg et al. (2000) prospectively examined the temporal relationship between stressful experiences and asthma in children, and showed that stressful life events increase the risk of a new asthma attack in the coming few weeks. Severe stress is associated with higher risk of self-reported asthma incidence in adults (Rod et al., 2012). Furthermore, psychological stress affects additional allergic diseases, including allergic rhinitis and food allergies (Rod et al., 2012; Heyman, 2005; Lebovidge et al., 2009).

Because young children have close ties with their parents, the psychological condition of parents may strongly influence their children. Wolf et al. (2008) reported that higher levels of perceived stress and depression in parents are associated with increases in IL-4 production and eosinophil cationic protein release in children. This pattern was observed in both asthmatic children and healthy children (Wolf, Miller & Chen, 2008). However, there have been no studies reporting a relationship between parental psychological stress and other biomarkers related to allergic conditions in children.

For children, inhalation and oral exposures are the main exposure routes of allergens, and this study focuses on them. Inhalation allergens, especially house dust mite (HDM), are mainly related to asthma and allergic rhinitis (Gandhi et al., 2013), and recent studies have reported that IL-22, predominantly secreted by T helper type 17 (Th17) cells, is highly expressed in various infectious and inflammatory diseases, especially childhood allergic rhinitis and asthma (Farfariello et al., 2011; Tsuji et al., 2012a). Polycyclic aromatic hydrocarbons (PAHs) are one of the causes of asthma related to air pollution, and Ple et al. (2015) recently found that PAH-induced IL-22 level in asthmatic patients is significantly higher than that in healthy subjects. IL-6 and IL-8 are pro-inflammatory cytokines, and many studies have confirmed increased IL-6 and IL-8 expressions in patients with asthma and allergic rhinitis (Shi et al., 2010; Tsuji et al., 2012b).

On the other hand, oral exposure is important, and a disruption of the intestinal barrier leads to food allergy (Jeon et al., 2013). Intestinal epithelial toll-like receptor (TLR) activation prevents food allergic responses, and the activated TLR increases IL-10/IL-13 expressions and suppresses IL-6 and IL-8 expressions (De Kivit et al., 2014). The deletion/anergy of reactive T cells against specific antigens are associated with the expansion of regulatory T cell population, and IL-10 production is mainly involved in oral tolerance (Crittenden & Bennett, 2005). Thus, in consideration of the main exposure routes of allergens among children, IL-6, IL-8, IL-10 and IL-22 were considered as proper biomarkers in this study.

For the evaluation of maternal psychological status, the Japanese Perceived Stress Scale (JPSS) and the Centre for Epidemiological Studies Depression Scale (CES-D) were used. The JPSS is a standardized self-reported questionnaire of globally perceived stress (Cohen, Kamarck & Mermelstein, 1983). The questions address how stressful, overwhelming, and uncontrollable a person has found his/her life during the last month. The CES-D is a short, self-reporting scale designed to measure depressive symptoms in the general population and clinical populations. The 20 symptom-related items of the CES-D examine how often one experienced depressive cognitions, affect, and behaviors during the past one week (Radloff, 1977).

The primary objective of this study is to test the usefulness of specific inflammatory biomarkers in the analysis of the potential relationship between diverse childhood allergies, including asthma, allergic rhinitis, and specific IgE reactions to HDM and food, and maternal psychological stress, as assessed using the JPSS and CES-D.

## Material and Methods

### Study subjects

In this study, children who were under the age of 4 and either of their parents were recruited at one clinic between January 2009 and February 2010 (Tsuji et al., 2012b; Tsuji et al., 2012a). We invited 203 pairs of mothers and children to participate in this study, and 158 of them (78%) agreed to participate. All participants were asked to return to the clinic when the child had been stable for more than one month without any clinical symptoms of allergic diseases. When they returned to the clinic, three questionnaires were administered to the mother. An initial questionnaire was given at the maternal interview, including questions about duration (weeks) of pregnancy, breastfeeding, maternal smoking habits, and allergy histories. Regarding maternal smoking habits, mothers who never smoked near their children were considered as non-smokers (Tsuji et al., 2012b; Tsuji et al., 2012a). After that, maternal psychological status was evaluated using two scales, the JPSS and CES-D, and 157 of them answered both the JPSS and CES-D. The remaining mother answered only the JPSS. Non-fasting venous blood was collected from children who were not infected with any diseases during the month preceding the interview. Children’s sera collected were subjected to the CAP radio-allergosorbent test (CAP-RAST) to examine responses to egg, milk, wheat and HDM. IL-6, IL-8, IL-10 and IL-22 mRNA were analyzed by quantitative reverse-transcriptase PCR (qRT-PCR) to estimate gene expression levels using the children’s blood samples. A blood sample for qRT-PCR was not collected from all of the 158 children. Pediatricians discontinued blood collection because children cried and/or their blood vessels were very thin and it was difficult to obtain sufficient blood volumes. Thus, the total number of study subjects was 152 in this study. The asthma group was defined by children who met at least two out of the three following criteria: (1) diagnosed by their primary physician as having asthma; (2) having experienced shortness of breath and/or wheezing; and (3) having received treatment for asthma or asthmatic bronchitis (Tsuji et al., 2012b; Tsuji et al., 2012a). The allergic rhinitis group was defined by children who met both of the following criteria: (1) diagnosed by an otolaryngologist as having allergic rhinitis; and (2) having experienced paroxysmal iterative sneezing, watery rhinorrhea and stuffy nose.

This study, including the manner in which written informed consent was obtained from the subjects’ guardians, was approved by the review boards of Kumamoto University (genome No 118) and the University of Occupational and Environmental Health (H23-03).

### Quantitative real-time PCR (q-RT-PCR) for cytokine mRNA expression

Blood samples were collected into heparin-coated vacuum tubes and kept at 4 °C until aliquots were transferred into RNA preparation solution within 6 h of collection. Total RNA was isolated from 185 µl of the whole blood of children using the QIAamp RNA Blood Mini kit (Qiagen, Hilden, Germany). RNA was reverse transcribed into cDNA using QuantiTect Reverse Transcription (Qiagen, Hilden, Germany) according to the manufacturer’s instructions and stored at −80 °C for qRT-PCR analysis. Quantitative detection of glyceraldehyde-3-phosphate dehydrogenase (GAPDH), IL-6, IL-8, IL-10 and IL-22 was performed using the StepOnePlus Real-Time PCR System (Applied Biosystems, Foster City, CA, USA) and the Fast SYBR Green Master Mix (Applied Biosystems, Foster City, CA, USA) according to the manufacturer’s protocol. GAPDH is one of the most commonly used housekeeping genes in comparisons of gene expression data. PCR amplification was carried out in a total volume of 20 µL containing 2 µL cDNA 10 µL and 0.2 µM of each primer. The PCR cycling conditions were 95°C for 5 min, followed by 55 cycles of 95°C for 10 s and 60°C for 30 s. To confirm the amplification specificity, we subjected all of the PCR products to melting curve analysis. The basic methodology of qRT-PCR was as described previously (Tsuji et al., 2012b; Vogel et al., 2005).

The primers for each gene were designed on the basis of the respective cDNA or mRNA sequences using OLIGO primer analysis software provided by Steve Rozen and the Whitehead Institute/MIT Center for Genome Research. Primers were as follows: for GAPDH: 5′-GAGTCAACGGATTTGGTCGT-3′ (forward), 5′-TTGATTTTGGAGGGATCTCG-3′ (reverse); for IL-6: 5′-GAACTCCTTCTCCACAAGCG-3′ (forward), 5′-TTTTCTGCCAGT GCCTCTTT-3′ (reverse); for IL-8: 5′-CTGCGCCAACACAGAAATTA-3′ (forward), 5′-ATTGCATCTGGCAACCCTAC-3′ (reverse); for IL-10: 5′-TGGGGGAGAACCTGAAGAC-3′ (forward), 5′-CCTTGCTCTTGTTTTCACAGG-3′ (reverse); and for IL-22: 5′-ACAGCAAATCCAGTTCTCCAA-3′ (forward), 5′-TCCAGAGGAATGTGCAAAAG-3′ (reverse).

### Allergen-specific IgE assays

Egg-, milk-, wheat-, and HDM-specific immunoglobulin E (IgE) was determined by the CAP-RAST using 0.3 ml of serum per allergen at FALCO Biosystems Ltd., in Japan. A result of 0.35 UA/ml was taken as a sign of sensitization, so 0.35 UA/ml or higher subjects were categorized as positive and lower than 0.35 UA/ml subjects were categorized as negative (Tsuji et al., 2012b).

### Statistical methods

The comparisons of CES-D and JPSS scores by the presence of allergic diseases and potential determinants were conducted by the Mann–Whitney *U* test or Kruskal–Wallis test. Each mRNA level of cytokine was applied in multivariable regression models as a dependent variable after log transformation. In multivariable analysis, age and sex of the study subjects were always included in the models because they are basic characteristics. In addition, the number of siblings was also included in the model based on the results of the association with parental psychological stress. All analyses were performed by STATA Version 10 (Stata Corporation, USA), and all *P* values presented are two-sided (*α* = 0.05).

## Results

Characteristics of the study subjects are summarized in Table 1. Median ages of children and their mothers were 13 months and 31 years old, respectively. Thirty-three mothers did not provide their age and one mother did not report gestational age at birth in the questionnaires. Three parents could not remember their child’s birth weight. HDM-specific IgE of 5 children could not be measured because of insufficient blood volume.

**Table 1:** Characteristics of the study population.

	Number/Median (%/25th and 75th percentiles)
**ALL**	152 (100%)
**Sex**	
Boy	83 (55%)
Girl	69 (45%)
**Age**	
Child (month)	13 (7, 26)
Mother (year)^a^	31 (29, 34)
**Gestational age (week)** ^a^	39 (38, 40)
**Birth weight (kg)** ^a^	3.0 (2.7, 3.3)
**Feeding**	
Breast	66 (43%)
Mix (breast + milk)	68 (45%)
Milk	18 (12%)
**Number of siblings**	
None	83 (55%)
1	54 (36%)
≥2	15 (10%)
**Smoking habits of parents**	
Smokers	86 (57%)
Non-smokers	66 (43%)
**Asthma**	
No	117 (77%)
Yes	35 (23%)
**Allergic rhinitis**	
No	134 (88%)
Yes	18 (12%)
**Food-specific IgE (egg and/or milk and/or wheat)**	
Negative	60 (39%)
Positive	92 (61%)
**HDM-specific IgE** ^a^	
Negative	118 (81%)
Positive	28 (19%)

**Notes.**

a The information of mother’s age, gestational age, birth weight, and HDM-specific IgE was unavailable in 33, 1, 3, and 5 subjects, respectively.

The distributions of maternal CES-D and JPSS scores were compared by the following variables: history of allergic diseases, the presence of specific IgE antibodies against foods (egg, milk or wheat) and HDM, age and sex of the children, mother’s age, the number of siblings, and smoking habits of parents. The maternal JPSS scores of the IgE-positive group for HDM were significantly higher than those of the negative group. A similar tendency was also observed in CES-D scores. The maternal CES-D scores of boys were significantly higher than those of girls (*P* = 0.012). There was a similar trend in JPSS scores and the difference was marginally significant (*P* = 0.083). Mothers with more than one child showed significantly higher JPSS scores than mothers with only one child (*P* = 0.011). A similar tendency was also observed in CES-D scores (Table 2).

**Table 2:** Comparison of CES-D and JPSS scores by potential determinants for allergic disease.

		CES-D				JPSS		
	*N*	Median	25th, 75th	*P* value^a^	*N*	Median	25th, 75th	*P* value^a^
**All**	150	8	3, 12		151	23	17, 27	
**Asthma**								
No	116	7	3, 12	0.355	117	23	17, 26	0.187
Yes	34	9	6, 12		34	25	19, 29	
**Allergic rhinitis**								
No	132	8	3 ,12	0.285	133	23	17, 27	0.691
Yes	18	10	6, 12		18	23	19, 27	
**Food-specific IgE**								
Negative	59	7	3, 12	0.739	59	23	18, 28	0.913
Positive^b^	91	8	4, 12		92	23	17, 27	
**HDM-specific IgE**								
Negative	117	7	3, 12	0.148	117	23	17, 26	0.034
Positive	28	9	5, 14		28	26	19, 31	
**Sex**								
Boy	83	9	5, 13	0.012	83	24	19, 28	0.083
Girl	67	6	1, 10		68	22	16, 26	
**Age of children (month)**								
<13	72	7	2, 12	0.365	73	23	17, 27	0.570
≥13	78	8	5, 12		78	24	18, 27	
**Age of mother (year)**								
<31	57	8	3, 12	0.512	59	23	16, 27	0.127
≥31	60	7	3, 11		59	22	17, 26	
Unknown	33	8	5, 12		33	26	20, 31	
**Number of siblings**								
0	81	7	3, 11	0.106	82	22	17, 26	0.011
≥1	69	9	4, 14		69	25	20, 30	
**Smoking habits of parents**								
Smokers	84	9	4, 14	0.100	86	23	19, 28	0.416
Non-smokers	66	7	3, 10		65	23	17, 26	

**Notes.**

a *P* value was obtained by Mann–Whitney *U* test except for age of mother. Kruskal–Wallis test was applied for age of mother.

b Egg and/or milk and/or wheat-specific IgE were positive.

The full scores of CES-D and JPSS were 60 and 56, respectively.

**Table 3:** Results of multivariable analysis in the relationships between CES-D and cytokine expressions by allergic disease.

	IL-6			IL-8			IL-10			IL-22		
CES-D	Coefficient	SE	*P* value^a^	Coefficient	SE	*P* value^a^	Coefficient	SE	*P* value^a^	Coefficient	SE	*P* value^a^
**All** (*N* = 150)	−0.011	0.013	0.393	−0.005	0.014	0.737	0.008	0.016	0.615	−0.016	0.024	0.503
**Asthma**												
No (*N* = 116)	−0.032	0.016	0.043	−0.024	0.017	0.168	−0.006	0.020	0.778	−0.044	0.029	0.134
Yes (*N* = 34)	0.045	0.022	0.048	0.037	0.024	0.143	0.043	0.030	0.153	0.043	0.039	0.276
**Allergic rhinitis**												
No (*N* = 132)	−0.018	0.014	0.182	−0.012	0.015	0.437	0.002	0.018	0.892	−0.021	0.026	0.421
Yes (*N* = 18)	0.072	0.055	0.217	0.045	0.041	0.274	0.065	0.044	0.169	−0.044	0.064	0.504
**Asthma and/or allergic rhinitis**												
No (*N* = 107)	−0.032	0.016	0.046	−0.021	0.018	0.236	−0.001	0.021	0.949	−0.045	0.030	0.137
Yes (*N* = 43)	0.043	0.025	0.092	0.033	0.024	0.170	0.031	0.027	0.244	0.043	0.038	0.258
**Food-specific IgE**												
Negative (*N* = 59)	−0.014	0.026	0.591	−0.013	0.023	0.583	−0.006	0.037	0.877	−0.011	0.047	0.824
Positive^b^ (*N* = 91)	−0.011	0.015	0.477	−0.003	0.186	0.859	0.011	0.017	0.527	−0.020	0.028	0.489
**HDM-specific IgE**												
Negative (*N* = 117)	−0.002	0.143	0.881	−0.022	0.017	0.192	0.003	0.020	0.900	−0.018	0.029	0.531
Positive (*N* = 28)	0.003	0.027	0.899	0.020	0.033	0.550	0.033	0.031	0.300	−0.055	0.048	0.265

**Notes.**

Coefficient regression coefficient SE standard error

a *P* value was obtained by multivariate regression models adjusted for sex, age, and number of siblings.

b Egg and/or milk and/or wheat specific IgE were positive.

**Table 4:** Results of multivariable analysis in the relationships between JPSS and cytokine expressions by allergic disease.

	IL-6			IL-8			IL-10			IL-22		
JPSS	Coefficient	SE	*P* value^a^	Coefficient	SE	*P* value^a^	Coefficient	SE	*P* value^a^	Coefficient	SE	*P* value^a^
**All** (*N* = 151)	−0.001	0.011	0.951	0.007	0.012	0.556	0.002	0.014	0.883	−0.005	0.021	0.794
**Asthma**												
No (*N* = 117)	−0.013	0.014	0.325	−0.011	0.015	0.476	−0.010	0.017	0.563	−0.024	0.025	0.347
Yes (*N* = 34)	0.029	0.019	0.141	0.052	0.022	0.023	0.026	0.026	0.320	0.029	0.033	0.391
**Allergic rhinitis**												
No (*N* = 133)	−0.009	0.012	0.441	0.001	0.013	0.945	−0.003	0.015	0.832	−0.012	0.022	0.598
Yes (*N* = 18)	0.115	0.045	0.023	0.063	0.036	0.104	0.055	0.042	0.216	0.013	0.061	0.837
**Asthma and/or allergic rhinitis**												
No (*N* = 108)	−0.018	0.013	0.195	−0.010	0.015	0.531	−0.010	0.018	0.564	−0.026	0.026	0.310
Yes (*N* = 43)	0.042	0.021	0.050	0.050	0.021	0.022	0.028	0.023	0.232	0.038	0.032	0.241
**Food-specific IgE**												
Negative (*N* = 58)	0.0028	0.024	0.908	0.005	0.022	0.825	0.002	0.035	0.948	−0.019	0.045	0.673
Positive^b^ (*N* = 91)	0.0006	0.013	0.964	0.005	0.015	0.733	−0.005	0.014	0.696	−0.001	0.023	0.960
**HDM-specific IgE**												
Negative (*N* = 116)	0.005	0.012	0.654	−0.013	0.014	0.379	−0.008	0.017	0.655	−0.012	0.025	0.638
Positive (*N* = 28)	0.017	0.025	0.484	0.033	0.030	0.275	0.033	0.028	0.247	−0.028	0.045	0.535

**Notes.**

Coefficient regression coefficient SE standard error

a *P* value was obtained by multivariate regression models adjusted for sex, age, and number of siblings.

b Egg and/or milk and/or wheat specific IgE were positive.

To clarify the effects of maternal psychological stress on their children, we examined the associations between CES-D/JPSS scores and the expression levels of four inflammatory biomarkers. The results of multivariable regression analyses are shown in Tables 3 and 4. The maternal CES-D scores were positively associated with IL-6 expression in children with asthma but not in those without allergic diseases. This interaction was statistically significant for IL-6 expression (*P* = 0.011 by likelihood ratio test). A similar tendency was also observed between subjects positive and negative for HDM-specific IgE, but there was no statistical significance. On the other hand, association or interaction with cytokine expressions was not observed for food-specific IgE (Table 3).

Regarding the JPSS, the score was positively associated with IL-8 expression among subjects with asthma and/or allergic rhinitis and there was marginally significant association with IL-6. However, subjects without these allergic diseases showed negative tendencies (Table 4). Additionally, there were significant interactions between JPSS score and the presence of asthma and allergic rhinitis regarding IL-6 (*P* = 0.017) and IL-8 (*P* = 0.034) expressions. The subjects positive for HDM-specific IgE were more likely to show positive associations between JPSS score and cytokine expressions than those negative for HDM-specific IgE. However, there was no statistical significance between them.

## Discussion

In this study, maternal CES-D score was positively associated with IL-6 expression in children with asthma, and maternal JPSS score was positively related to IL-6 and IL-8 expressions among children with asthma and/or allergic rhinitis. They were not so strongly correlated, but the results are interesting. Recently, Shi et al. (2010) found that the protease-activated receptor (PAR)/phosphatidylinositol 3 kinase (PI3K)/NF*κ*B signaling pathway is involved in the induction of IL-6 and IL-8 by *Dermatophagoides pteronyssinus* 1 (Der p1) in airway epithelial cells. Der p1 is one of the major protease allergens of HDM, and is a crucial aeroallergen and an independent risk factor for asthma and/or allergic rhinitis (Huss et al., 2001). The PAR/PI3K/NF*κ*B signaling pathway by Der p1 might be activated by the stress of caregivers. As a result, IL-6 and IL-8 might have increased in our study. Certainly, if this pathway is really related to our results, the relationship of the stress of caregivers with IL-8 and IL-6 would have been stronger in the HDM-specific IgE-positive group. However, the subjects were young children. Generally, HDM-specific IgE increases after the age of three (Ng, Holt & Prescott, 2002). The age of our subjects might have influenced our results.

Since the psychological status of young children cannot be evaluated, the question is whether there is a correlation between the psychological stress of a mother and her child. A recent cohort study, Growing Up in Ireland, National Longitudinal Study of Children, reported that babies’ development could be affected by stress or depression among their parents (Nixon, Swords & Murray, 2013). This finding suggested the possibility that maternal stress could influence the immune system of children. Since the immune responses of allergic children are more sensitive than those of healthy children, the increases of IL-6 and -8 expressions could be detected in their study. In order to support our research, further experiments *in vitro* and *in vivo* are required to clarify the relationship between psychological stress, allergy, and PAR/PI3K/NF*κ*B signaling pathway.

In this study, both maternal perceived stress and depression showed similar tendencies in the association with cytokine expression patterns by the presence of allergic diseases (Tables 3 and 4). Although there was a significant correlation between CES-D and JPSS scores, Spearman’s rho: 0.78, *P* = 0.001 (data not shown), the associations between JPSS and cytokine expressions were relatively evident. Perceived stress is slightly different from depression psychologically. Since there is a strong relationship between high stress level and depression (Wiegner et al., 2015), it is difficult to distinguish between them (Van Praag, 2004). Furthermore, the difference in the score distribution between CES-D and JPSS could explain the evident results in JPSS. The JPSS score distribution was close to normal distribution with a median of 23 (56 points = full score). However, the CES-D score distribution was skewed to the right with a median of 8 (60 points = full score), and this might be one of the reasons for the weak association with CES-D. Tomljenovic, Pinter & Kalogjera (2014) reported that chronic psychological stress might be one of the factors that modifies disease severity and may lead to uncontrolled diseases in chronic rhinosinusitis patients. Patterson et al. investigated whether participants’ reporting of allergy flares correlated with perceived emotional stress, depression, mood, and a biomarker of stress. They reported that the allergy flare group had higher perceived stress scores than those of the group without allergy symptoms. Perceived stress, but not depressive symptoms, is positively correlated with allergy flares (Patterson et al., 2014). Considering the findings of these studies, allergic diseases might be more related to perceived stress than depression.

Our results were interesting in that IL-6 and IL-8 were related to physiological stress and childhood allergies. IL-6 is a T helper type 2 (Th2) cytokine. Th2 cells produce other various cytokines, such as IL-4, IL-5 and IL-13 (Fallon et al., 2002). IL-1*β* potently stimulates IL-8 expression (Liu et al., 2014). We should measure such cytokines in the future to confirm our results.

In this study, the asthmatic group included subjects with a combination of criteria: diagnosis by a doctor, symptoms and receipt of treatment. However, diagnosing asthma in pre-school children is difficult. Spirometry and lung function parameters could support the clinical diagnosis of asthma, but most techniques depend on the effort and cooperation of the patient and thus not applicable in pre-school children (Kooi et al., 2006). Therefore, there is the possibility that children without asthma might have been included in the asthma group in this study.

In conclusion, the most important finding from this study is that there are significant relationships between maternal psychological stress and IL-6 and IL-8 mRNA expressions in children with asthma and allergic rhinitis.

## Supplemental Information

10.7717/peerj.1585/supp-1 Supplemental Information 1 Stata command

10.7717/peerj.1585/supp-2 Supplemental Information 2 Raw data

## Additional Information and Declarations

### Data Availability

The following information was supplied regarding data availability: The data set and commands are available as Supplemental Information.
