# Supplementary material for: The association between maternal psychological stress and inflammatory cytokines in allergic young children
Source: PeerJ. 2016 Jan 18;4:e1585. doi: 10.7717/peerj.1585 (PMC4727978; doi:10.7717/peerj.1585)
Supplement: Supplemental Information 1 [file peerj-04-1585-s001.zip › PeerJ stata command.docx]

**excluded subjects without information cesd and IL

drop if il6==.

drop if cesd==. & pss==.

*drop if cesd==.

gene age_gp=1 if age>=0 & age<=6

replace age_gp=2 if age>=7 & age<=12

replace age_gp=3 if age>=13 & age<=18

replace age_gp=4 if age>=19 & age<=24

replace age_gp=5 if age>=25

gene age_gp3=1 if age>=0 & age<10

replace age_gp3=2 if age>=10 & age<20

replace age_gp3=3 if age>=20 & age<=100

gene age_gp2=1 if age>=0 & age<13

replace age_gp2=2 if age>=13 & age<=100

gene mage_gp2=1 if agemother>=0 & agemother<31

replace mage_gp2=2 if agemother>=31 & agemother<=60

replace mage_gp2=3 if agemother==.

gene lil6=ln(il6)

gene lil8=ln(il8)

gene lil10=ln(il10)

gene lil22=ln(il22)

gene lcox2=ln(cox2)

gene lfoxp3=ln(foxp3)

gene lsocs3=ln(socs3)

gene lrelb=ln(relb)

mean cesd

mean pss

gene cesd_gp=1 if cesd>=0 & cesd<8.3

replace cesd_gp=2 if cesd>=8.3

gene pss_gp=1 if pss>=0 & pss<22.5

replace pss_gp=2 if pss>=22.5

tab cesd_gp

tab pss_gp

gene il8_gp=1 if cesd<1.95

replace il8_gp=2 if cesd>=1.95

gene il6_gp=1 if cesd<3.08

replace il6_gp=2 if cesd>=3.08

gene crean_gp=1 if creaner<=2

replace crean_gp=2 if creaner==3

replace crean_gp=3 if creaner==4

replace crean_gp=4 if creaner>=5

tab crean_gp

gene crean_grp=1 if creaner<=3

replace crean_grp=2 if creaner==4

replace crean_grp=3 if creaner==5

tab crean_grp

gene bedtime_gp=1 if bedtime==1

replace bedtime_gp=1 if bedtime==2

gene asthma=1 if asthmadiagnosis==2

replace asthma=2 if asthmadiagnosis==1

gene ar=1 if allergicrhinitis==2

replace ar=2 if allergicrhinitis==1

gene sib_gp=0 if brother==1

replace sib_gp=1 if brother==2

replace sib_gp=3 if brother==3 | brother==4

gene sib_gp2=0 if brother==1

replace sib_gp2=1 if brother>=2 & brother<10

****************************************

****************************************

****************************************

***診ood IgE No******

gene rast1ageno3_gp=1 if rast1ageno3==0

replace rast1ageno3_gp=2 if rast1ageno3>=1

replace rast1ageno3_gp=. if rast1ageno3==.

tab rast1ageno3_gp

**HDM IgE**********

gene dani_gp=1 if dustclass==0

replace dani_gp=2 if dustclass>=1

replace dani_gp=. if dustclass==.

**Table1**************************************

tab sex

centile age , centile (25, 50, 75)

centile agemother, centile (25, 50, 75)

centile pregnancy, centile (25, 50, 75)

centile weight0m, centile (25, 50, 75)

tab milk

tab sib_gp

tab smoking

tab asthma

tab ar

tab rast1ageno3_gp

tab dani_gp

**Table2 ***1219****************************************************

* CESD and JPSS & potential confounding factor

****************************************************************

centile cesd pss, centile (25, 50, 75)

by asthma, sort: centile cesd pss, centile (25, 50, 75)

by ar, sort: centile cesd pss, centile (25, 50, 75)

by rast1ageno3_gp, sort: centile cesd pss, centile (25, 50, 75)

by dani_gp, sort: centile cesd pss, centile (25, 50, 75)

by sex, sort: centile cesd pss, centile (25, 50, 75)

by age_gp2, sort: centile cesd pss, centile (25, 50, 75)

by mage_gp2, sort: centile cesd pss, centile (25, 50, 75)

by sib_gp, sort: centile cesd pss, centile (25, 50, 75)

by sib_gp2, sort: centile cesd pss, centile (25, 50, 75)

by smoking, sort: centile cesd pss, centile (25, 50, 75)

ranksum cesd, by(asthma)

ranksum cesd, by(ar)

ranksum cesd, by(rast1ageno3_gp)

ranksum cesd, by(dani_gp)

ranksum cesd, by(sex)

ranksum cesd, by(age_gp2)

kwallis cesd, by(mage_gp2)

kwallis cesd, by(sib_gp)

kwallis cesd, by(sib_gp2)

ranksum cesd, by(smoking)

ranksum pss, by(asthma)

ranksum pss, by(ar)

ranksum pss, by(rast1ageno3_gp)

ranksum pss, by(dani_gp)

ranksum pss, by(sex)

ranksum pss, by(age_gp2)

kwallis pss, by(mage_gp2)

kwallis pss, by(sib_gp)

ranksum pss, by(sib_gp2)

ranksum pss, by(smoking)

********Table4****1219************************************

tab asthmadiagnosis allergicrhinitis if cesd>=0 & cesd<50

**喘息・鼻炎なし１、どちらか２、すべてあり３**

gene allergy_g=1 if asthma==1 & ar==1

replace allergy_g=2 if asthma==1 & ar==2

replace allergy_g=2 if asthma==2 & ar==1

replace allergy_g=3 if asthma==2 & ar==2

tab allergy_g if cesd>=0 & cesd<50

**喘息・鼻炎なし1、どちらか少なくともある2。*acta pediatrica　使用*

gene asthmaar=1 if allergy_g==1

replace asthmaar=2 if allergy_g>=2

**

**喘息・鼻炎・食物全部なし1、どれかあり2、すべてあり3**

gene allergy_grp=1 if asthma==1 & ar==1 & rast1ageno3_gp==1

replace allergy_grp=2 if asthma==1 & ar==1 & rast1ageno3_gp==2

replace allergy_grp=2 if asthma==1 & ar==2 & rast1ageno3_gp==1

replace allergy_grp=2 if asthma==2 & ar==1 & rast1ageno3_gp==1

replace allergy_grp=2 if asthma==1 & ar==2 & rast1ageno3_gp==2

replace allergy_grp=2 if asthma==2 & ar==1 & rast1ageno3_gp==2

replace allergy_grp=2 if asthma==2 & ar==2 & rast1ageno3_gp==1

replace allergy_grp=3 if asthma==2 & ar==2 & rast1ageno3_gp==2

tab allergy_grp if cesd>=0 & cesd<50

**CESD*****************

**Table3-1*****

**Asthma*

*il6*

xi:reg lil6 cesd i.sex age brother if cesd>=0 & cesd<50

xi:reg lil6 cesd i.sex age brother if asthma==1 & cesd>=0 & cesd<50

xi:reg lil6 cesd i.sex age brother if asthma==2 & cesd>=0 & cesd<50

xi:reg lil6 i.asthma*cesd i.sex age brother if cesd>=0 & cesd<50

est store a

xi:reg lil6 i.asthma cesd i.sex age brother if cesd>=0 & cesd<50

est store b

lrtest a b

*il8*

xi:reg lil8 cesd i.sex age brother if cesd>=0 & cesd<50

xi:reg lil8 cesd i.sex age brother if asthma==1 & cesd>=0 & cesd<50

xi:reg lil8 cesd i.sex age brother if asthma==2 & cesd>=0 & cesd<50

xi:reg lil8 i.asthma*cesd i.sex age brother if cesd>=0 & cesd<50

est store a

xi:reg lil8 i.asthma cesd i.sex age brother if cesd>=0 & cesd<50

est store b

lrtest a b

*il10*

xi:reg lil10 cesd i.sex age brother if cesd>=0 & cesd<50

xi:reg lil10 cesd i.sex age brother if asthma==1 & cesd>=0 & cesd<50

xi:reg lil10 cesd i.sex age brother if asthma==2 & cesd>=0 & cesd<50

xi:reg lil10 i.asthma*cesd i.sex age brother if cesd>=0 & cesd<50

est store a

xi:reg lil10 i.asthma cesd i.sex age brother if cesd>=0 & cesd<50

est store b

lrtest a b

*il22*

xi:reg lil22 cesd i.sex age brother if cesd>=0 & cesd<50

xi:reg lil22 cesd i.sex age brother if asthma==1 & cesd>=0 & cesd<50

xi:reg lil22 cesd i.sex age brother if asthma==2 & cesd>=0 & cesd<50

xi:reg lil22 i.asthma*cesd i.sex age brother if cesd>=0 & cesd<50

est store a

xi:reg lil22 i.asthma cesd i.sex age brother if cesd>=0 & cesd<50

est store b

lrtest a b

**

***AR*****

*il6*

xi:reg lil6 cesd i.sex age brother if ar==1 & cesd>=0 & cesd<50

xi:reg lil6 cesd i.sex age brother if ar==2 & cesd>=0 & cesd<50

xi:reg lil6 i.ar*cesd i.sex age brother if cesd>=0 & cesd<50

est store a

xi:reg lil6 i.ar cesd i.sex age brother if cesd>=0 & cesd<50

est store b

lrtest a b

*il8*

xi:reg lil8 cesd i.sex age brother if ar==1 & cesd>=0 & cesd<50

xi:reg lil8 cesd i.sex age brother if ar==2 & cesd>=0 & cesd<50

*il10*

xi:reg lil10 cesd i.sex age brother if ar==1 & cesd>=0 & cesd<50

xi:reg lil10 cesd i.sex age brother if ar==2 & cesd>=0 & cesd<50

*il22*

xi:reg lil22 cesd i.sex age brother if ar==1 & cesd>=0 & cesd<50

xi:reg lil22 cesd i.sex age brother if ar==2 & cesd>=0 & cesd<50

**Asthma and or AR*****

xi:reg lil6 cesd i.sex age brother if asthmaar==2 & cesd>=0 & cesd<50

xi:reg lil6 cesd i.sex age brother if asthmaar==1 & cesd>=0 & cesd<50

xi:reg lil6 i.asthmaar*cesd i.sex age brother if cesd>=0 & cesd<50

est store a

xi:reg lil6 i.asthmaar cesd i.sex age brother if cesd>=0 & cesd<50

est store b

lrtest a b

**

xi:reg lil8 cesd i.sex age brother if asthmaar==2 & cesd>=0 & cesd<50

xi:reg lil8 cesd i.sex age brother if asthmaar==1 & cesd>=0 & cesd<50

xi:reg lil8 i.asthmaar*cesd i.sex age brother if cesd>=0 & cesd<50

est store a

xi:reg lil8 i.asthmaar cesd i.sex age brother if cesd>=0 & cesd<50

est store b

lrtest a b

**

xi:reg lil10 cesd i.sex age brother if asthmaar==1 & cesd>=0 & cesd<50

xi:reg lil10 cesd i.sex age brother if asthmaar==2 & cesd>=0 & cesd<50

**

xi:reg lil22 cesd i.sex age brother if asthmaar==1 & cesd>=0 & cesd<50

xi:reg lil22 cesd i.sex age brother if asthmaar==2 & cesd>=0 & cesd<50

*****Food IgE******

*il6*

xi:reg lil6 cesd i.sex age brother if rast1ageno3_gp==1 & cesd>=0 & cesd<50

xi:reg lil6 cesd i.sex age brother if rast1ageno3_gp==2 & cesd>=0 & cesd<50

*il8*

xi:reg lil8 cesd i.sex age brother if rast1ageno3_gp==1 & cesd>=0 & cesd<50

xi:reg lil8 cesd i.sex age brother if rast1ageno3_gp==2 & cesd>=0 & cesd<50

*il10*

xi:reg lil10 cesd i.sex age brother if rast1ageno3_gp==1 & cesd>=0 & cesd<50

xi:reg lil10 cesd i.sex age brother if rast1ageno3_gp==2 & cesd>=0 & cesd<50

*il22*

xi:reg lil22 cesd i.sex age brother if rast1ageno3_gp==1 & cesd>=0 & cesd<50

xi:reg lil22 cesd i.sex age brother if rast1ageno3_gp==2 & cesd>=0 & cesd<50

***HDM dani IgE***

*il6*

xi:reg lil6 cesd i.sex age brother if dani_gp==1 & cesd>=0 & cesd<50

xi:reg lil6 cesd i.sex age brother if dani_gp==2 & cesd>=0 & cesd<50

*il8*

xi:reg lil8 cesd i.sex age brother if dani_gp==1 & cesd>=0 & cesd<50

xi:reg lil8 cesd i.sex age brother if dani_gp==2 & cesd>=0 & cesd<50

*il10*

xi:reg lil10 cesd i.sex age brother if dani_gp==1 & cesd>=0 & cesd<50

xi:reg lil10 cesd i.sex age brother if dani_gp==2 & cesd>=0 & cesd<50

*il22*

xi:reg lil22 cesd i.sex age brother if dani_gp==1 & cesd>=0 & cesd<50

xi:reg lil22 cesd i.sex age brother if dani_gp==2 & cesd>=0 & cesd<50

**Table3-2***PSS******************

**Asthma**************

*il6*

xi:reg lil6 pss i.sex age brother

xi:reg lil6 pss i.sex age brother if asthma==1

xi:reg lil6 pss i.sex age brother if asthma==2

xi:reg lil6 i.asthma*pss i.sex age brother

est store a

xi:reg lil6 i.asthma pss i.sex age brother

est store b

lrtest a b

*il8*

xi:reg lil8 pss i.sex age brother

xi:reg lil8 pss i.sex age brother if asthma==1

xi:reg lil8 pss i.sex age brother if asthma==2

xi:reg lil8 i.asthma*pss i.sex age brother

est store a

xi:reg lil8 i.asthma pss i.sex age brother

est store b

lrtest a b

*il10*

xi:reg lil10 pss i.sex age brother

xi:reg lil10 pss i.sex age brother if asthma==1

xi:reg lil10 pss i.sex age brother if asthma==2

*il22*

xi:reg lil22 pss i.sex age brother

xi:reg lil22 pss i.sex age brother if asthma==1

xi:reg lil22 pss i.sex age brother if asthma==2

**

***AR****

*il6*

xi:reg lil6 pss i.sex age brother if ar==1

xi:reg lil6 pss i.sex age brother if ar==2

xi:reg lil6 i.ar*pss i.sex age brother

est store a

xi:reg lil6 i.ar pss i.sex age brother

est store b

lrtest a b

*il8*

xi:reg lil8 pss i.sex age brother if ar==1

xi:reg lil8 pss i.sex age brother if ar==2

xi:reg lil8 i.ar*pss i.sex age brother

est store a

xi:reg lil8 i.ar pss i.sex age brother

est store b

lrtest a b

*il10*

xi:reg lil10 pss i.sex age brother if ar==1

xi:reg lil10 pss i.sex age brother if ar==2

xi:reg lil10 i.ar*pss i.sex age brother

est store a

xi:reg lil10 i.ar pss i.sex age brother

est store b

lrtest a b

*il22*

xi:reg lil22 pss i.sex age brother if ar==1

xi:reg lil22 pss i.sex age brother if ar==2

**Asthma and or AR***

xi:reg lil6 pss i.sex age brother if asthmaar==1

xi:reg lil6 pss i.sex age brother if asthmaar==2

xi:reg lil6 i.asthmaar*pss i.sex age brother

est store a

xi:reg lil6 i.asthmaar pss i.sex age brother

est store b

lrtest a b

**

xi:reg lil8 pss i.sex age brother if asthmaar==1

xi:reg lil8 pss i.sex age brother if asthmaar==2

xi:reg lil8 i.asthmaar*pss i.sex age brother

est store a

xi:reg lil8 i.asthmaar pss i.sex age brother

est store b

lrtest a b

**

xi:reg lil10 pss i.sex age brother if asthmaar==1

xi:reg lil10 pss i.sex age brother if asthmaar==2

xi:reg lil10 i.asthmaar*pss i.sex age brother

est store a

xi:reg lil10 i.asthmaar pss i.sex age brother

est store b

lrtest a b

**

xi:reg lil22 pss i.sex age brother if asthmaar==1

xi:reg lil22 pss i.sex age brother if asthmaar==2

xi:reg lil22 i.asthmaar*pss i.sex age brother

est store a

xi:reg lil22 i.asthmaar pss i.sex age brother

est store b

lrtest a b

*****Food IgE******

*il6*

xi:reg lil6 pss i.sex age brother if rast1ageno3_gp==1 & cesd>=0 & cesd<50

xi:reg lil6 pss i.sex age brother if rast1ageno3_gp==2 & cesd>=0 & cesd<50

*il8*

xi:reg lil8 pss i.sex age brother if rast1ageno3_gp==1 & cesd>=0 & cesd<50

xi:reg lil8 pss i.sex age brother if rast1ageno3_gp==2 & cesd>=0 & cesd<50

*il10*

xi:reg lil10 pss i.sex age brother if rast1ageno3_gp==1 & cesd>=0 & cesd<50

xi:reg lil10 pss i.sex age brother if rast1ageno3_gp==2 & cesd>=0 & cesd<50

*il22*

xi:reg lil22 pss i.sex age brother if rast1ageno3_gp==1 & cesd>=0 & cesd<50

xi:reg lil22 pss i.sex age brother if rast1ageno3_gp==2 & cesd>=0 & cesd<50

***HDM dani IgE***

*il6*

xi:reg lil6 pss i.sex age brother if dani_gp==1 & cesd>=0 & cesd<50

xi:reg lil6 pss i.sex age brother if dani_gp==2 & cesd>=0 & cesd<50

*il8*

xi:reg lil8 pss i.sex age brother if dani_gp==1 & cesd>=0 & cesd<50

xi:reg lil8 pss i.sex age brother if dani_gp==2 & cesd>=0 & cesd<50

*il10*

xi:reg lil10 pss i.sex age brother if dani_gp==1 & cesd>=0 & cesd<50

xi:reg lil10 pss i.sex age brother if dani_gp==2 & cesd>=0 & cesd<50

*il22*

xi:reg lil22 pss i.sex age brother if dani_gp==1 & cesd>=0 & cesd<50

xi:reg lil22 pss i.sex age brother if dani_gp==2 & cesd>=0 & cesd<50
