# Supplementary material for: The association between maternal psychological stress and inflammatory cytokines in allergic young children
Source: PeerJ. 2016 Jan 18;4:e1585. doi: 10.7717/peerj.1585 (PMC4727978; doi:10.7717/peerj.1585)
Supplement: Supplemental Information 2 [file peerj-04-1585-s002.zip › Raw data peerJ.pdf]

NO	性	casecontrol			「皮膚」<br>37.発疹6ヶ	43.アレルギー性鼻炎	採血1回目	採血2回目
NO	sex	casecontrol	Asthma diagnosis	symptom	eruption	allergicrhir	age	age2
1	1		2	2	1	2	11	
2	1		2	1	1	2	18	
3	2		2	1	1	1	28	
4	2		2	1	1	2	29	
5	1		2	1	1	2	22	
6	1		2	3	2	1	7	
7	2		2	1	2	2	14	20
8	2		2	1	2	2	21	
9	1		2	1	1	1	29	
10	1		2	2	2	2	36	
11	1		2	2	2	1	10	
12	1		2	2	2	2	19	
13	2	1	1	3	1	2	66	
14	1		1	2	2	2	10	
15	1	2	2	0	2	2	18	
16	1	1	1	1	1	2	35	
17	1		2	1	1	2	19	
18	2	1	1	3	2	2	31	
19	2	1	2	2	2	2	23	
20	2	2	2	0	2	1	23	
21	1		2	0	2	2	29	
22	2	2	2	0	2	2	7	12
23	1		2	0	2	2	25	
24	2		2	2	1	2	39	
25	1	1	2	2	1	2	11	
26	1		2	0	1	2	14	
27	1		2	0	1	2	6	10
28	1		2	1	2	2	22	
29	2		2	2	2	2	7	
30	2	2	2	0	2	2	8	
31	2	2	2	0	2	2	38	
32	1		2	2	2	1	6	
33	1		2	3	2	1	35	
34	1	2	2	0	2	2	19	
35	2	2	2	0	2	2	12	
36	1	1	1	3	2	2	47	
37	1		1	0	1	2	15	
38	2		2	2	1	2	57	
39	1		1	1	1	1	52	
40	1		2	2	2	2	6	11
41	2		1	0	2	2	14	
42	2	2	2	0	1	2	6	
43	2	2	2	0	2	2	9	16
44	2	2	2	0	2	2	27	
45	2		2	3	2	2	28	
46	2	2	2	0	1	2	13	
47	1		2	2	2	2	3	6
48	2		1	2	1	1	77	
49	2	1	2	2	1	2	11	19
50	1	2	2	0	2	1	39	
51	2		2	3	2	2	21	
52	2	2	2	0	2	2	28	
53	1	2	2	0	2	1	18	
54	1		2	2	2	2	13	
55	2	2	2	0	2	2	6	

56	1	2	2	0	2	2	6	
57	1		2	1	1	2	15	
58	1		2	3	2	2	9	
59	1		1	3	2	2	33	
60	2	2	2	0	2	2	12	20
61	2		2	1	1	2	29	
62	1	2	2	0	2	2	21	
63	2		2	3	1	2	26	
64	1		2	1	2	2	66	
65	1	2	2	0	1	2	40	
66	1		1	3	1	1	13	
67	1		2	2	2	2	8	
68	2	2	2	0	1	2	30	
69	1		2	1	2	2	6	
70	2	2	2	0	2	2	5	9
71	1		2	2	2	2	72	
72	1		2	3	2	2	11	
73	1	2	2	0	2	2	7	
74	2		2	3	1	2	44	51
75	1		2	1	2	2	13	
76	2	1	1	3	2	2	29	
77	1	2	2	1	1	2	39	
78	1		2	3	1	2	8	
79	1		1	0	2	2	40	48
80	2	2	2	1	2	2	5	12
81	2		2	1	2	2	5	
82	1		2	1	2	2	30	
83	1	1	2	3	2	2	30	
84	1		1	3	1	1	27	
85	2	2	2	0	2	2	26	
86	2	2	2	0	2	2	6	
87	2	2	2	0	2	2	6	12
88	2		1	1	2	1	17	
89	1	2	2	0	1	2	6	
90	1	2	2	0	2	2	7	
91	1	2	2	0	2	2	6	
92	2	2	2	1	2	2	39	
93	1		2	2	2	2	15	
94	1	1	1	3	1	1	15	21
95	1		1	1	1	2	18	
96	1	2	2	0	2	2	3	9
97	1	2	2	1	2	1	12	
98	2		2	1	1	1	8	
99	2		2	1	1	2	36	
100	2		1	1	2	2	14	
101	1		2	1	2	2	6	
102	2	2	2	0	1	2	13	
103	2	1	1	3	2	2	29	
104	1		2	3	2	2	6	10
105	1	2	2	0	2	2	6	
106	2	2	2	0	2	2	6	
107	1	2	2	0	1	2	16	22
108	1	1	1	3	2	1	29	36
109	1	2	2	0	2	2	3	
110	1	1	1	3	2	2	11	
111	2	1	1	3	1	2	37	
112	2		2	1	2	2	8	
113	1	1	1	3	1	1	48	
114	2	2	2	0	2	2	11	

115	2		1	0	2	2	14	
116	1	2	2	0	1	2	10	16
117	1	1	2	2	1	1	7	
118	1	2	2	0	1	2	29	
119	1	2	2	0	1	2	7	
120	1	2	2	0	2	2	6	
121	1	1	1	3	1	1	35	43
122	1		1	3	1	2	9	
123	2		2	1	2	2	7	
124	1		2	1	1	2	13	
125	2	2	2	0	2	2	9	
126	2	2	2	0	1	2	7	11
127	1		2	2	1	2	16	
128	2	1	1	3	1	1	31	
129	1		2	3	2	2	17	
130	1		1	3	1	2	31	
131	1		1	1	1	2	8	
132	1		2	3	2	2	20	
133	1		2	1	2	2	6	
134	1		2	1	2	2	6	
135	1	1	1	3	1	2	19	
136	1	1	1	3	2	2	24	
137	2	2	2	0	1	2	7	
138	1		1	2	1	2	19	
139	2		2	1	1	2	7	
140	1	2	2	0	1	2	14	
141	2		2	2	1	2	7	12
142	1	2	2	0	2	2	8	
143	1		2	1	2	2	23	
144	1		2	1	2	2	12	
145	2		2	1	2	2	23	
146	2		1	1	1	2	30	
147	1		2	3	2	2	10	
148	1		1	3	1	1	72	
149	2	2	2	0	2	2	6	10
150	2	1	1	1	2	2	23	29
151	2	2	2	0	2	2	6	
152	2	2	2	0	2	2	7	12
153	2		2	3	1	1	10	
154	2		2	3	2	2	22	
155	1	2	2	0	1	2	7	12
156	2		2	2	2	2	10	
157	2	2	2	0	2	2	7	
158	2	2	2	0	1	2	7	11
159	1		1	3	1	1	14	
160	1		1	3	2	2	10	
161	2	2	2	0	2	2	13	
162	2	2	2	0	1	2	14	
163	2		1	3	1	2	9	15
164	1	2	2	0	2	2	47	
165	2	2	2	0	2	2	7	12
166	2	2	2	0	2	2	18	
167	2		2	2	1	2	16	
168	2	1	1	3	1	2	22	
169	2	2	2	0	1	2	48	
170	1		2	3	1	2	7	
171	1	2	2	0	2	2	17	25
172	2	2	2	1	1	2	32	
173	1	2	2	0	1	2	27	

174	2		2	1	1	2	60	
175	1	2	2	0	1	2	72	
176	2		1	1	2	2	24	
177	2	2	2	0	2	2	15	
178	2	1	1	3	2	2	28	
179	1	1	1	3	1	1	11	
180	1	2	2	0	1	1	36	
181	1	2	2	0	2	2	8	
182	2	1	1	3	2	1	48	
183	1	1	2	3	2	1	24	
184	2	2	2	0	1	2	60	
185	2	1	1	3	1	2	36	
186	1	2	2	0	2	2	11	
187	1		2	1	1	2	9	
188	1	2	2	0	1	2	6	10
189	2	2	2	0	2	2	7	
190	2	2	2	0	2	2	15	
191	1	1	2	2	1	2	12	
192	2	2	2	0	1	2	9	
193	1	2	2	0	2	2	7	
194	2	2	2	0	2	2	9	
195	1	1	1	3	2	2	9	
196	2		1	3	2	2	29	
197	1	2	2	0	2	2	21	
198	1	2	2	0	2	2	21	
199	1	2	2	0	2	2	10	
200	1		2	2	1	2	6	
201	2	2	2	0	2	2	11	
202	2	1	1	3	2	2	19	
203								
204								
205								
206								
207								
208								
209								
210								
211								
212								
213								
214								
215								
216								
217								

採血3回目RAST陽性		授乳形態	兄弟番目					cesd
age3	rast1agenc	milk	brother	IL22	IL10	IL8	IL6	cesd
	1	2	2	27.85981	3.577669	0.238697	3.241829	
	3	2	1	8.316953	5.023734	0.729504	4.128119	
	1	2	2	30.80245	1.09228	0.742025	1.639479	
	1	2	1	85.38692	2.18052	1.596516	1.001957	3
	2	1	1	8.255703	0.774164	1.894218	0.870143	7
	0	1	1	8.537947	1.530748	0.757077	0.516847	12
26	1	1	1	11.62147	1.588605	0.003132	1.229889	12
	0	1	2	8.401918	2.933393	0.30411	0.630665	3
	2	2	1	2.624194	2.718898	0.152448	0.412258	12
	0	2	2	5.706536	1.551143	2.738117	0.621161	
	0	1	2	1.239717	1.859421	1.424506	3.055403	
	0	3	3	1.018813	0.657516	0.451177	0.57345	18
	0	2	1	9.580591	0.54317	3.681722	0.301421	5
	2	1	2	67.53521	2.542656	0.892406	1.73323	11
	0	2	1	1	1	1	1	11
	3	2	1	53.4746	2.57703	3.471141	0.669084	9
	0	2	2	64.34709	2.075939	1.244957	0.702397	15
	2	2	2	3.525004	4.061677	0.255241	1.307807	9
	0	1	2	1.33476	3.223907	1.516128	0.521241	10
	0	1	4					10
	1	1	1	2.576717	1.00382	0.965464	0.271981	12
	0	1	2	3.441244	2.529015	1.03453	0.445373	12
	1	3	1	35.25792	3.027941	1.165801	0.996731	16
	1	2	1	4.409525	0.374065	1.811012	0.338879	13
	0	2	2	24.18404	1.640635	0.751538	0.538453	
	0	3	1	5.099383	1.775557	1.054499	0.4478	14
	1	1	1.0	2.83766	0.836573	0.775835	1.015496	9
	1	2	1	5.433217	1.678354	0.050897	0.291601	
	0	2	3	37.06203	1.288449	0.429362	0.407735	
	0	2	2	82.53564	1.528109	2.062374	0.752629	2
	0	2	1	0.945983	1.531657	2.403065	0.776668	0
	0	2	2	5.078493	7.390088	0.319579	0.65196	
	0	2	1	21.68801	0.689877	1.305328	0.398735	13
	1	2	1	59.17724	3.103257	0.696104	0.62682	9
	0	2	1	3.899397	1.933719	0.691599	0.564198	5
	3	1	3	1.080785	1.844318	0.488967	0.765297	
	1	2	3	2.53142	1.891568	3.026708	0.505224	6
	0	1	3	71.30623	2.734481	0.096012	2.010121	0
	0	3	1	104.1546	1.010208	0.769706	0.237303	7
17	1	2	2	4.991216	2.090915	2.108022	1.292535	4
	0	3	2	21.85781	18.90378	3.278027	0.954047	16
	1	1	1	1.581631	1.550847	1.728447	0.418288	9
	0	2	1	2.48217	1.122818	0.951141	1.719267	9
	0	1	1	1.029841	4.131466	0.54695	0.354946	5
	0	2	2	3018.416	0.416978	0.021637	18.04647	14
	0	2	2	56.18774	1.323122	0.634621	2.248083	20
	0	1	2	120.6034	1.347555	1.552677	1.600605	19
	0	2	1					3
	0	1	3	53.01115	2.166795	1.705025	0.900324	1
	1	2	1	67.37157	3.459848	1.4549	3.760466	6
	0	1	1	53.69854	1.557616	4.028664	1.900223	
	1	2	1	75.40476	0.649901	2.004707	1.501484	
	0	2	1	50.40866	1.906544	1.019059	1.062662	12
	1	2	1	120.4379	9.690309	0.289624	5.709426	8
	0	1	2					8

	0	2		1	88.34873	7.506171	3.165752	4.01621	19
	1	2		2					
	3	1		2	53.33141	1.171256	2.936365	2.219027	13
	2	1		2	23.79175	2.53222	3.129229	1.235262	0
	0	2		2	56.48977	5.437191	0.941445	8.71075	3
	0	2		2	235.3506	3.302587	1.657952	4.53923	13
	2	2		1	94.94997	1.476178	1.120128	3.388464	5
	0	2	1.0		39.84851	3.159618	2.233809	2.193122	1
	0	1		1	320.5386	2.631114	5.451097	3.108694	7
	0	1		2	123.0478	0.836314	6.795905	2.189637	7
	2	1	1.0		52.20623	1.703035	2.096476	3.984635	
	0	2		2	11.41988	8.976796	1.799221	1.331945	4
	2	2		1	92.23721	1.532477	3.502618	2.213524	10
	0	2		2	484.2471	2.842334	0.619053	6.361945	0
	2	1		2	426.7636	1.578433	1.603356	8.997185	12
	0	1		1					3
	1	1		2	110.0511	1.944956	1.365438	1.37983	6
	0	2		1	96.46937	9.176079	1.743583	3.53702	4
	3	2		1	20.8069	2.950647	3.893128	1.240857	
	1	2		1	1.17454	7.548326	1.64263	1.919853	6
	1	2		1	35.64096	9.465531	0.574151	2.033085	
	3	1		1	42.1495	0.471524	0.977448	1.006932	
	1	2		2	6.050664	2.193574	1.313074	1.917989	
	3	1		1	2.616507	9.712023	0.580764	1.70449	
	2	2		2	78.77831	2.566313	0.509253	1.258623	22
	1	2		3	71.3265	11.54402	1.004811	2.996465	22
	2	2		1	193.9669	9.103952	4.136478	5.773735	5
	3	2		2	103.7509	2.359622	3.14531	0.917414	5
	2	2		3	3.564369	20.91982	1.07614	3.242094	
	0	2		1	0.765909	3.332742	0.819204	0.735327	8
	1	1		1	4.855126	6.245704	1.312283	5.446234	12
	0	2		1	7.022084	8.205041	1.798733	9.50012	7
	0	2		2	162.2484	9.950952	2.208651	1.315929	
	2	1		1	348.7674	4.600163	1.34866	0.701631	16
	2	2		1	288.0965	2.963145	2.809948	6.0342	7
	0	2		2	37.92404	6.036083	0.994202	5.148666	9
	2	2		1	140.2248	2.506162	3.75745	7.119207	
	2	2		2	10.4714		2.145195	3.483356	
	1	2		3	9.700948	3.294724	0.243444	1.715521	10
	1	2		3	64.98778	14.00488	5.333529	6.911704	29
	1	2		2	544.6601	19.84413	1.306806	8.326998	
	0	1		2	35.99797	3.811626	1.385896	13.32943	11
	3	1		1					
	2	3		3	23.03395	3.925207	5.16813	5.654955	
	1	1		1	11.76318	7.872167	0.14068	1.803016	
	0	1		1	35.05953	8.641515	1.641857	1.931074	0
	0	2		2	46.3819	13.56042	1.155336	2.294551	10
	3	1		3	49.3965	3.407309	1.823414	2.348931	8
15	1	2		1	54.50686	9.210451	1.398793	4.518644	5
	1	2		2	36.70281	4.36637	1.185161	1.830943	
	0	1		2	75.46121	16.65285	0.58549	4.261868	8
	1	1		1	54.58409	27.01109	2.70946	5.267281	
	1	1		2	83.93913	0.177595	1.528625	1.256485	1
	0	2		3	66.13685	4.645542	0.767902	1.505776	14
	0	1		2	28.77045	15.97694	1.553207	11.72847	10
	1	1		1	26.14947	2.168852	0.991421	1.307508	19
	0	2		2	30.48184	8.392293	0.85882	1.548446	22
	0	2		2	67.4921	10.0738	2.075517	1.289855	
	1	3		2	161.4398	5.258806	1.372801	0.027563	20

0	2	1	123.1748	19.95652	14.42302	1.127745	
1	2	2	41.8914	13.08194	0.367965	2.523367	1
3	1	2	21.63857	1.867717	0.892818	1.376349	11
3	1	2	17.79322	0.438304	1.280452	1.550466	21
2	1	1	38.16093	4.932505	3.790916	2.31306	10
0	2	3	60.02905	2.91882	2.227692	1.36086	8
2	1	1	308.2595	2.468285	1.588036	1.444023	8
1	2	3	13.39907	19.23181	4.410329	4.636584	
2	2	2	238.7247	7.128135	1.874353	5.593803	3
2	1	1	108.298	20.82482	2.739598	3.57366	16
1	1	3	85.90869	7.285175	21.98266	13.10405	12
2	2	4	202.5604	3.160675	2.190413	4.362175	8
1	1	3	60.6177	4.172442	3.236001	4.813645	7
1	2	1	260.3229	1.504419	2.019134	2.783798	7
0	2	1	97.91323	30.26268	4.128818	3.749282	11
0	2	2	204.1412	18.39219	4.341021	6.429683	12
2	1	1	386.413	5.936774	3.569571	4.030849	9
1	1	1	144.2249	8.540465	2.807211	1.784672	
0	2	2	5.83285	7.342223	2.045773	0.593648	
2	1	2	5.164318	8.280867	2.442936	1.516691	34
3	3	2	12.02918	8.278689	3.058988	1.356148	6
3	3	1	30.58935	13.55447	3.880477	0.926046	
1	1	1	88.93774	10.65218	6.958327	4.624389	3
1	2	1	21.47513	1.792594	3.295682	0.975134	8
1	1	1	73.90488	6.227943	0.606389	2.172353	0
1	1	1	63.0249	12.38338	3.580266	2.858584	5
3	2	1	72.76905	4.00935	4.64292	5.134052	
0	2	1	53.05181	7.468349	0.306059	4.32146	15
3	1	1	11.59183	5.279391	3.695845	1.03277	1
1	1	2	1.710262	12.46022	0.673735	12.90138	9
0	1	1	11.23181	6.39299	4.071828	3.016119	1
1	3	1	4.16893	2.406457	2.736402	0.694721	9
2	1	2	71.66205	7.723716	7.335189	5.306774	8
3	2	1	23.68339	2.58536	4.971406	1.958565	20
1	1	1	32.52114	3.364938	0.432069	0.529159	1
2	2	1	154.1465	2.701909	2.463952	2.258958	9
1	1	1	655.3904	7.49344	1.995557	1.323356	0
1	1	1	0.439047	2.207498	8.94669	3.205971	0
0	2	2	6.821631	7.293202	0.608551	0.68983	5
2	2	1	30.00889	13.5041	0.921953	0.848756	6
1	2	1	30.39351	1.778395	0.575549	1.193528	1
0	3	2	25.98474	10.66091	1.825138	5.911602	1
2	1	1	86.66479	1.840099	1.068421	6.80351	0
2	1	1	179.5081	10.27926	3.10414	4.725388	0
3	1	1	29.33366	8.662058	3.989717	7.708353	22
1	2	1	205.7311	17.52617	3.8995	13.64665	14
1	1	1					0
1	1	1					2
3	1	2	25.90112	17.32494	0.420105	8.032462	4
2	1	1	70.30295	11.99937	0.977478	1.67701	10
3	1	1	9.459076	24.89508	0.536141	4.734981	
2	1	1					9
1	3	4	285.8436	11.00916	1.121911	1.102034	5
1	2	1	2.477884	18.97363	0.165949	4.755225	1
0	3	1	127.5969	7.263958	2.378018	4.037574	3
2	3	1					12
2	1	3	9.289623	5.165083	1.066703	2.499398	12
0	2	1	17.47303	0.000257	0.602965	1.137289	3
1	1	2	4.154273		2.030841	2.124973	

0	1	1	6.320564	3.72686	1.122367	1.210924	
2	1	3	41.46086	10.09659	3.130396	5.094807	
0	1	2	33.05195	2.920341	0.923315	1.743332	1
2	1	2	512.8746	1.579905	1.214122	4.225423	0
1	3	1	5.646759	3.775845	0.695377	1.233094	
0	3	2	7.131255	4.365804	0.357831	3.0507	1
0	1	2	6.160669	4.304929	1.711427	0.342949	2
1	1	2	37.06101	4.935264	2.001809	2.120645	1
2	1	1	100.2392	4.802445	4.096764	2.398767	10
2	1	2	326.2208	1.913678	6.892514	6.677303	
0	1	1	1797.558	0.551829	6.197421	5.065082	
3	2	1	168.5024	1.552562	1.758192	1.948694	6
0	3	1	463.4292	1.230015	0.657075	2.457835	1
2	1	2	394.8133	0.211321	1.524374	1.78764	6
1	3	1	714.9153	2.562892	0.418095	5.513407	3
0	1	1	4418.914	1.948402	0.350719	18.55808	6
1	1	3	2834.364	1.800896	3.640435	9.945192	
1	2	1	313.3306	2.091742	2.539871	4.543655	7
0	1	1	2554.753	1.297479	0.436335	11.96786	7
0	3	2	35.45518	2.18339	0.138711	1.141241	15
2	2	1	486.016	0.596291	1.509518	1.809839	0
2	3	2	433.0828	0.400284	0.289951	2.712248	21
1	3	1	461.2647	1.083702	0.91506	4.059498	12
1	3	2	144.6245	5.67547	0.208867	3.02223	12
1	1	2	600.4881	0.721145	0.414315	1.483754	2
3	1	1	403.0843	0.912399	0.993482	5.377051	2
2	1	2	882.4075	0.869745	0.488502	2.645722	14
1	1	1	61.69504	4.471831	2.112868	2.684462	6
0	2	1	323.9995	0.695874	0.981158	1.459493	0

PSS	検査時年	卵1	牛乳1	小麦1	ピーナツ1	大豆1	米1	ゴマ1
PSS	rast1age	egg1	milk1	wheat1	peanut1	soybean1	rice1	sesame1
	11	0.85	0	0	0	0	0	0
	9	4.09	5.69	0	0	0	0	0
	6	3	0	0	0	0	0	0
27	11	3	0	0	0	0	0	0
21	6	0.47	0	0.5	0	0	0	0
17	7	0	0	0	0	0	0	0
26	9	0						
26	21	0	0	0	0	0	0	0
26	6	3	0	1		0	0	
	7	2	0	0	0	0	0	0
	10	0	0	0	0	0	0	0
34	19	0	0	0	0	0	0	0
	66	0	0	0	0	0	0	0
24	6	0.35	0	0	0	0	0	0
28	6	0	0	0	0	0	0	0
19	5	5	0	3		2		0
26	19	0	0	0	0	0	0	0
26	8	0	0	0	0	0	0	0
17	8	0	0	0	0	0	0	0
34	23	0	0	0	0	0	0	0
25	7	0	0	0	0	0	0	0
34	7	0	0	0	0	0	0	0
34	6	1.28	0	0	0	0	0	0
35	39	0.7	0	0	0	0	0	0
	2	0	0	0		0	0	
31	7	0	0	0	0	0	0	0
22	6	7.83	0	0	0	0	0	0
	15	0.58	0	0	0	0	0	0
	7	0	0	0	0	0	0	0
18	8	0	0	0	0	0	0	0
15	26	0.47	0	0	0	0	0	0
	6	0	0	0	0	0	0	0
23	12	0.4	0	0	0	0	0	0
17	8	28.2	0	0	0	0	0	0
24	12	0	0	0	0	0	0	0
	38	28.4	100	4.71	0.37	0.42		6.53
28	6	1.26	0	0	0	0	0	0
16	57	0	0	0	0	0	0	0
19	52	0	0	0	0	0	0	0
19	6	1.75	0	0	0	0	0	0
26	7	0	0	0	0	0	0	0
23	6	2.06	0	0	0	0	0	0
19	9	0	0	0	0	0	0	0
24	11	0.91	0.74	2.38	2.22	0.82	0	3.92
26	28	0	0	0	0	0	0	0
31	7	0	0	0	0	0	0	0
29	3	0	0	0	0	0		0
19	19	2	1	0	0	0	0	0
23	7	0	0	0	0	0	0	0
23	39	1.26	0	0	0	0	0	0
	7	0	0	0	0	0	0	0
	28	0.41	0	0	0	0	0	0
28	18	0	0	0	0	0	0	0
23	8	2.35	0.65	0	0	0	0	0
28	6	0	0	0	0	0	0	0

29	6	0	0	0	0	0	0	0
	6	0	1.23	0	0	0	0	0
30	9	19.34	8.65	0.47	0	0.93	0	0
9	7	11.1	0.35	0	0.7	0	0	0
22	12	0	0	0	0	0	0	0
31	29	0	0	0	0	0	0	0
20	21	5.86	1.3	0	1.77	0	0	9.68
17	26	0	0	0	0	0	0	0
26	66	0	0	0	0	0	0	0
26	40	0	0	0	0	0	0	0
	13	2.32	0	0.59	0.5	0.35	0.48	0.63
17	8	0	0	0	0	0	0	0
23	12	3.72	4.62	0	8.85	0.53	0	0.83
22	6	0	0	0	0	0	0	0
26	5	1.05	2.22	0	0	0	0	0
8	72	0	0	0	0	0	0	0
25	7	1.43	0	0	0	0	0	0
25	7	0	0	0	0	0	0	0
	6	33	3.06	20		0.81	5.45	
16	7	10.4	0	0	0	0	0	0
	29	5.58	0	0	0	0	0	0
	2	0	0	0		0		
	8	0.56	0	0	0	0	0	0
	13	37.5	19.9	2.55	5.6	6.23	0.64	3.31
35	5	2.16	0.37	0	0	0	0	0
35	5	0	0.41	0	0	0	0	0
19	7	5.91	1.08	1.08	0	0	0	0
19	7	30.8	16.6	12.9	0	0	0	0.38
	27	0.86	0	0.52	0	0	0	0
20	26	0	0	0	0	0	0	0
29	6	4.91	0	0	0	0	0	0
19	6	0	0	0	0	0	0	0
	17	0	0	0	0	0	0	0
26	6	8.63	0	2.19	0	0	0.41	0
22	7	17.5	5.11	0	1	0.81	0	0.4
24	6	0	0	0	0	0	0	0
	6	20.3	0	0	0	0	0	0
	7	0.8	0	0	0	0	0	0
22	15	3.24	0	0	0	0	0	0
37	8	3	0	0				
	3	1.01	0	0	0	0	0	0
31	6	0	0	0	0	0	0	0
	8	16.9	7.22	34.4	0.67	1.9	0.97	14.3
	36	0.91	0.72	0	0	0	0	0
	14	0.39	0	0	0	0	0.47	0
12	6	0	0	0	0	0	0	0
24	6	0	0	0	0	0	0	0
26	6	5.43	0	0	0	0	0	0
26	6	1.13	0	0	0	0	0	0
	6	1.38	0	0	0	0	0	0
20	6	0	0	0	0	0	0	0
	6	3(スコアの	0	0	0			
20	23	8.14	0	1.12	0	0	0	0.35
31	3	0	0	0	0	0	0	0
39	11	0	0	0	0	0	0	0
29	3	9.95	0	0		0	0	
29	8	0	0	0	0	0	0	0
	48	0	0	0	0	0	0	0
30	11	1.84	0	0	1.6	0	0	0

29	14	0	0	0	0	0	0	0
24	6	6.4	0	0.38	0	0	0	0
21	5	3(スコアの	0	0	0	0	0	0
36	7	6.19	0.44	0		0	0	
26	7	19.3	1.83	0	0	2.09	0	0
28	6	0	0	0	0	0	0	0
19	11	31.1	2.17	20.2	0.66	1.38	0	0
	6	7.26	4.86	4.28				
25	7	15.5	0.48	0	0	0	0	0
42	8	8.39	0.8	0	0	0	0	0
35	9	0.36	0	0	0	0	0	0
23	7	19.6	6.17	0	0	0	0	0
15	9	5.86	0	0	0	0	0	0
26	16	1.2	0	0	2.09	0	0.74	
23	9	0	0	0	0	0	0	0
27	31	0	0	0	0	0	0	0
27	8	11.3	15.2	0	0	0	0	0
	20	0.51	0	0	0	0	0	0
	3	0	0	0	0	0	0	0
26	6	18.2	0	0.76	0	0	0	0
16	19	0.94	0.65	0.67	0.51	0	0.39	1.65
	24	2.28	5.28	0.58	0	0	0	0
13	7	1.79	0	0	0	0	0	0
32	7	0	0.46	0	0	0	0	0
3	7	2.2	0	0	0	0	0	0
16	14	0.62	0	0	0	0	0	0.99
19	7	57.3	0.88	3.42	0	3.52	0	0
21	8	0	0	0	0	0	0	0
12	5	66.1	2.86	2.05	42.4	0	0	0
26	7	2.12	0	0	0	0	0	0
12	13	0.4	0	0	0	0	0	0
23	30	0.42	0	0	0	0	0	0
23	6	0	1.79	0	0	0	0	0
27	72	2(スコアの	2	2				
10	6	1.6	0	0	0	0	0	0
35	8	3.29	0	0	0	0	0	0
10	6	6.61	0	0	0	0	0	0
7	7	1.49	0	0	0	0	0	0
22	10	0	0	0	0	0	0	0
18	22	3.23	1.17	0	0	0	0	0.49
14	7	0.77	0	0	0	0	0	0
11	10	0	0	0	0	0	0	0
10	7	4.38	1.94	0	0	0	0	0
17	7	11.6	0.48	0	0	0	0	9.07
37	9	1.38	0.63	0	0	0	0	0
23	10	0.54	0	0	0	0	0	0
9	8	1.08	0	0	0	0	0	0
15	8	2.43	0.4	0	0	0	0	0
21	6	0.59	0	14.5	0	0	0	0
25	47	1.02	0	1.53	1.99	1.37	1.84	1.96
	7	13.7	0.38	0	0	0.61	0	0
25	18	0.61	0	0.71	0.42	0	0	0.42
8	16	0.36	0	0	0	0	0	0
10	22	1.95	0	0	0	0	0	0
20	48	0	0	0	0	0	0	0
26	7	17.4	0	6.78	0	1.45	2.41	0.35
26	7	2.07	0	0	0	0	0	0
17	13	2.66	0	0	0	0	0	0
	27	2.11	0	0	2.23	0.36	0	0.58

	60	0	0	0	0	0	0	0
	72	0.72		0.42				
10	24	0	0	0	0	0	0	0
7	3	21.7	0.84	0	0	0	0	
	10	1.34	0	0	0	0	0	0
13	11	0	0	0	0	0	0	0
19	36	0	0	0	0	0	0	0
12	8	10.4	0	0	0	0	0	0
30	4	10.9	2.39					
	11	0	0.35	0.42	0	0.4	0	0
	60	0	0	0	0	0.49	0	0
21	36	0.54	0.49	2.65	0	0	0	1.26
10	11	0	0	0	0	0	0	0
30	9	11.5	0	3.37	0	0	0	0
11	6	0.82	0	0	0	0	0	0
20	7	0	0	0	0	0	0	0
	15	0.76	0	0	0	0	0	0
23	7	4.17	0	0	0	0	0	0
19	9	0	0	0	0	0	0.44	0
27	7	0	0	0	0	0	0	0
15	9	5.15	0	0.52	0	0	0	0
30	9	0.6	0.61	0	0	0	0	0
24	9	0.43	0	0	0	0	0	0
24	6	0.66	0	0	0	0	0	0
11	13	1.67	0	0	0	0	0	0
14	10	42.6	14.3	1.28	0	0.42	4.88	4.54
36	6	92.2	0	1.05	0	0	0	0
16	7	0.56	0	0	3.11	0	0	0.8
10	9	2(スコアの	0	0		0		

そば1	えび1	いわし1	ハウスダスト1	ダニ1	総IgE1	検査時年	卵2	牛乳2
buckwheat	shrimp1	sardine1	housedust1	mite1	IgE1	agerast2	egg2	milk2
0	0	0	0	0	0			
0	0	0				13	5.49	2.99
0	0	0	0	0		15	1.23	0
0	0	0		0		29	0.95	0
0	0	0		0		13	3.8	0
0	0	0		0				
					14	14	6.33	0
0	0	0		0				
	0		0	0	20	11	4	
0	0	0				14	1	0
0	0	0		0	53			
0	0	0		0				
0	0	0	0	0				
0	0	0		0		10	0.76	0.63
0	0	0		0		11	0.41	0
				0	132	7	3	3
0	0	0		0	16			
0	0	0		0		31	0.87	0.38
0	0	0		0		17	1.2	0
0	0	0		0	31			
0	0	0		0		13	0	0
0	0	0	0	0	35	12	0	0
0	0	0				10	2.17	0
0	0	0		0	23			
					15	11	7.68	0
0	0	0		0		14	0	0
0	0	0		0	23	10	10.5	0.87
0	0	0		0		22	0.48	0
0	0	0		0	54			
0	0	0		0	16			
0	0	0	0	0		38	0	0
0	0	0		0	7			
0	0	0	0	0		35	0	0
0	0	0		0		19	5.24	0
0	0	0		0	69			
	0.42	1.66		100		47	13.9	48.1
0	0	0		0		15	0.68	0
0	0	0	6.44	6.49	136			
0	0	0		0				
0	0	0		0	23	11	4.57	0
0	0	0		0		14	0	0
0	0	0		0	17			
0	0	0		0		16	0	0
0	0	0	0	0		16	0.51	0
0	0	0		0				
0	0	0		0		13	0	0
						6	0	0
0	0	0	5	5		35	1	2
0	0	0		0	52	11	1.18	0
0	0	0		0				
0	0	0		0		14	0.76	0
0	0	0		0				
0	0	0		0	6			
0	0	0		0		13	1.36	0
0	0	0			5			

0	0	0		0	18			
0	0	0	0	0		10	21.2	3.29
0	0	0		0	520	13	29	7.67
0	0	0				11	8.79	0.5
0	0	0		0	13	20	0.64	0.45
0	0	0		0	24			
0.8	0	2.08		0				
0	0	0		0.59				
0	0		12.7	13.3	57			
0	0	0	0	0	19			
0.4	0	0		52.4	230			
0	0	0		0	3			
0.42	0	0				30	1.43	1.39
0	0	0		0	12			
0	0	0		0	56	9	4.78	1.96
0	0.67		0.73	0.6	40			
0	0	0		0		11	0.5	0
0	0	0		0	3			
					158	13	56.1	4.41
0	0	0	0	0		13	3.83	0
0	0	0		0				
				0	34	15	100	2.99
0	0	0			26			
0	1.07	2.26		72		29	19.7	12.9
0	0	0			15	12	2.96	6.19
0	0	0		0	6			
0	0	0				18	1.42	0
0	0	0				18	15	5.26
0	0	0		57.8	195			
0	0	0	0	0	8			
0	0	0		0	17	11	2.42	0
0	0	0			6	12	0	0
0	0	0	0	0				
0	0	0			42	10	10.9	0.63
0	0	0			126	11	9.48	1.83
0	0	0		0	5			
0	0	0			53	18	9.06	0
0	0	0		0		15	0	0
0	0	0		0	145	21	0.95	0
						18	2.61	0
0	0	0		0	7	9	1.04	0
0	0	0		0		12	0	0
0	2.38	0			383	12	8.75	6.3
0	0	0		33.1	226			
0	0	0		0	9			
0	0	0		0	6	12	0	0
0	0	0		0		13	2.18	0.41
0	0	0				12	3.69	0
0	0	0		0	36	10	11.5	1.2
0	0	0		0	20			
0	0	0		0	11			
				0	30	16	6.29	0
0	0	0			5	29	1.65	0
0	0	0		0	10			
0	0	0		0				
	0		0	0	28.8	13	12.8	
0	0	0		0	13			
0	0	0		0				
0	0	0		2.79	40	18	0.87	0.77

0	0	0			20			
0	0	0	0	0	122	10	4.72	0
			0	0	38	7	100	58.8
	0		0	0	97	17	5.19	0
0	0	0		0	100	11	18.3	1.59
0	0	0		0	8	13	0.43	0
0		0	0	0	229	19	28	0.69
				0		9	5.32	0
0	0	0		0	59	12	6.58	0
0	0	0		0		13	5.26	0.47
0	0	0			35	13	0	0
0	0	0			142	11	20.6	6.27
0	0	0		0		16	2.49	0
0				21.1		23	0.86	0
0	0	0		0		17	0	0
0	0	0		0	121			
0	0	0		0	76	12	6.42	6.73
0	0	0		0	134			
0	0	0			2	6	0	0
0	0.9	0		0	146			
0	0	0			684			
0	0	0		0	288			
0	0	0		0	14			
0	0	0		0		11	1.83	0
0	0	0			12	12	6.42	0
0.6	0	0.53						
0	0	0	0	0	250	12	33.2	1.64
0	0	0		0	11			
0	0	0		0		9	13.9	22.1
0	0	0		0		12	0.87	0
0	0	0		0		23	0	0
0	0	0			969			
0	0	0		0	13	10	2.53	1.75
	1.12		9.28	10.3	367			
0	0	0			30	10	4.56	0
0	0	0	0	0		13	5.97	1.66
0	0	0		0	20			
0	0	0		0	24	12	2.49	0
0	0	0		0	12			
0	0	0		100	374			
0	0	0		0	11	12	0.95	0
0	0	0		0	5			
0	0	0			30	11	2.64	0.78
0	0	0			101	11	12.2	0.58
0	0	0		0		14	1.3	0.56
0	0	0		0	12	15	0	0
0	0	0			13	13	0.91	0
0	0	0		0		14	2.19	0
0	0	0				9	1.82	0.39
1.71	0	0		33.7	112			
0	0	0		0	57	12	9.3	0.84
0	0	0			70			
0	0	0		0	198			
0	0	0			94			
0	0	0	0	0	13			
0	0	0			171	11	11	0
0	0	0		0		17	0.54	0.38
0	0	0	0	0		32	0	0
0	0	0		39.2	168			

0	0	0	3.8	4.01	187			
			45.5	45.1	138			
0	0	0		0	42			
						10	4.44	2.4
0	0	0	0	0		15	0.51	0
0	0	0		0	10			
0	0	0		0				
0	0	0		0	30			
						18	39.1	0.35
0	0	0		0		24	0	0.69
0	0	0	0	0	34			
0	0	0		17.1	169			
0	0	0	0	0	11			
0	0	0		0	111	13	6.52	0
0	0	0	0	0	7	10	0.69	0
0	0	0	0	0	40			
0	0	0	0	0	53			
0	0	0		33.2		12	1.59	0
0	0	0	0		85			
0	0	0	0		25			
0.68	0	0	0		22			
0	0	0	0	0	59			
0	0	0	0	0		29	0.69	0
0	0	0		0		21	0.65	0
0	0	0		0		21	1.54	0
0.35	0	0		0	316	13	24.9	7.15
0	0	0	0	0	797	9	82.4	0
0	0	0	0		9	11	0.38	0
					16	14		

小麦2	ピーナツ2	大豆2	米2	ゴマ2	そば2	えび2	いわし2	ハウスダスト2
wheat2	peanut2	soybean2	rice2	sesame2	buckwheat	shrimp2	sardine2	housedust2
0	0	0	0	6.31	0	0	0	
0	0	0	0	0	0	0	0	
0	0	0	0	0	0	0	0	
1.52	0	0	1.12	0	0	0	0	
0	0	0	0	0	0	0	0	
4				0				2
0	0	0	0	0	0	0	0	
0	0	0	0	0	0	0	0	0
0	0	0	0	0	0	0	0	
3		3	0	0	0			
0	0	0	0	0	0	0	0	0
0	0	0	0	0	0	0	0	
0	0	0	0	0	0	1.18	0	
0	0	0	0	0	0	0	0	0
0	0	0	0	0	0	0	0	
1.48	0	0.4	0	1.78	0	15	0	
0	0	0	0	0	0	0	0	
0.38	0	0	0	0.37	0	0	0	
0	0	0	0	0	0	0	0	
0	0	0		0		0		
0	0	0		0		0		0.6
0	0	0	0	0	0	0.54	0	
2.17	0	0	0	3.2		0	0.75	
0	0	0	0	0	0	0	0	
0	0	0	0	0	0	0	0	
0	0	0	0	0	0	0	0	
0	0	0	0	0	0	0	0	0
1.05	0.6	0.49	0	2.41	0	0	0	
0	0	0	0	0	0	0	0	
0	0	0	0	0	0	0	0	
0	0	0	0	0	0	0	0	6
0	0	0	0	0	0	0	0	
0	0	0	0	0	0	0	0	
0.45	0	0	0	0	0	0	0	

0.8	0	0	0	0	0	0	0.38	
1.14	0.54	3.99	0	0.57	0.37	0	0	
0	2.09	0		0	0	0	0	
0	0	0	0	0	0	0	0	
0	4.52	0	0	0	0	0	0	
0.43	0	0	0	0	0	0	0	
0	0	0	0	0	0	0	0	
68.7	3.25	22.2	43.6	42.9	7.58	0	6.23	
0	0	0	0	0	0	0	0	0
3.46	78.2	12.7		22.4	1.74			
2.52	3.37	3.6	0.82	2.89		0	0.36	
0	1.61	0	0	0	0	0	0	
2.74	0	0	0	0	0	0	0	
7.93	0	0	0	0	0	0	0	
0.59	0	0	0	0	0	0	0	
0	0	0	0	0	0	0	0	
1.3	0	0	0	0	0	0	0	
0	1.51	0	0	0.59	0	0	0	
2.98	3.95	2.56		5.33				2.55
0	0	0	0	0	0	0	0	
0	0	0	0	0	0	0	0	
0	0	0	0	0	0	0	0	
0	0	0	0	0	0	0	0	
0	0	0	0	0	0	0	0	0
13.5	2.31	1.58	1.02	40.5		4.86		
0	0	0	0	0	0	0	0	
0	0	0	0	0	0	0	0	
0.41	1.03	0	0	2.25	0	0	0	
0	2.13	0	0	0	0	0	0	
0	0	0	0	1.49	0	0	0	0
0	0	0	0	0	0	0	0	0
0.49	1.44	0	0	0	0	0	0	

0	0	0	0	0	0	0	0	0
1.7	2.23	0	0	0	0	0	0	
0		0	0	0	0	0		
4.34	0.43	3.31	0	5.28	1.43	0	0	
0	0	0	0	0	0	0	0	
6.31	0.44	0.47	0	0	0	0	0	
3.9	0	1.35	0	1.26	0	0	0	
0	0	0	0	0	0	0	0	
0	0	0	0	0	0	0	0	
0	0	0	0	0	0	0	0	
0	0	0	0	0	0	0	0	0
0	0	0	0	0	0	0	0	0
0	0.99	0	0.44	0	0	0	0	100
0	0	0	0	0	0	0	0	
0	0	0	0	0	0	0	0	
0	0	0	0	0	0	0	0	
0	0	0	0	0	0	0	0	
0	0	0	0	0	0	0	0	
1.65	0	4.67	0	0.9	0.6	0	0	
6.55	17.5	0.78	0	5.4				
0	0	0	0	0	0	0	0	
0	0	0	0	0	0	0	0	
0	0	0	0	0	0	0	0	
0	0	0	0	0	0	0	0	
0	0	0	0	0	0	0	0	
0	0	0	0	0	0	0	0	
0	0	0	0	0	0	0	0	
0	0	0	0	0	0	1.17	0	
0	0	0	0	5.21	0	0	0	
0.54	0	0	0	0	0	0	0	
0	0	0	0	0	0	0	0	
0	0	0	0	0	0	0	0	
0	0	0	0	0	0	0	0	
13.7	0	0.57	0	5.27	0.65	0	0	
0.65	0	3.3	0	0.43	0	0	0	
5.86	0.38	4.89	4.98	1.42	0.88	0	0	
0	0	0	0	0	0	0	0	
0	0	0	0	0		0		0

2.19	3.23	0	0	0	0	0	0	
0	0	0	0	0	0	0	0	
0.46	0	0	0	0	0	0	0	5.53
0.49	0	0	0	0	0	0	0	
0	0	0	0	0	0	0	0	
0	0	0	0	0	0	0	0	12
0	0	0	0	0	0	0	0	
0	0	0	0	0	0	0	0	0
0	0	0	0	0	0	0	0	
0.62	0	0	1.46	2.75	0	0	0	
1.38	0	0	0	0				
0	0	0	0	0	0	0	0	0

ダニ2<br>mite2	総IgE2<br>IgE2	検査時年<br>agerast3	卵3<br>egg3	牛乳3<br>milk3	小麦3<br>wheat3	ピーナツ3<br>peanut3	大豆3<br>soybean3	米3<br>rice3
0		18	2.78	3.23	0	0	0	
0		28	0	0	0	0	0	0
0	60							
0		22	0.73	0	0.6	0	0	0.39
0		20	2.21	0	0	0	0	0
0	212	20	5		5			
0		36	0	0	0	0	0	
	39							
0		18	0	0	0	0	0	0
		11	5	2	6		4	
0	63							
0		23	0	0	0	0	0	0
1.24		29	0.53	0	0	0	0	0
0	71							
0		17	1.36	0	0	0	0	0
68.8	268							
3.83	17							
0.99								
0	8							
0.58	16							
100								
0	22							
	40	17	6.77	0	0	0	0	0
0	2							
	7							
7.27		27	0	0	0	0	0	0
0								
0	6							
5		57	0	0.42	0	0	0	0
0	81	19	0	0	0	0	0	0
0		21	0.45	0	0	0	0	0
0								

		15	16.9	1.42	0	0	0	0
	1214							
0		24	4.17	0	0	0.63	0	
0								
0								
0	56							
		29	100	5.73	100	3.07	15	39.8
0	0	21	2.01	0	0	0	0	0
0		27	68.3	0.93		19.5		12
		40	6.43	10.1	1	1.77	1.23	0.5
21.7	1342							
5.38		24	1.27	0	1	0	0	0
20.6		24	20.8	13.4	12.2	0	0	0
0								
2.92		24	13.4	1.05	6.49	5.65	5.49	7.83
0								
0								
	18							
0	10							
0	4							
0								
0	63	20	1.4	0.82	0	0	0	0
		29	26.8	0.53	0.8	0	0	0
0		15	14	1.65	0	1.57	0	0
0		22	1.25	0	0	0	0	0
0.99		36	0.91	0	0	0	0	0
		23	9.87					
10.1								

0		16	0.96	0	0	0	0	0
	1275	11	100	23.6	27	26	5.33	1
		29	2.88	0.66	0.77	0.45	0.45	0.53
0		29	22	0.56	3.25	4.83	0	
0								
	127							
1.25								
100		31	0.56	0	0	0.7	0	0.42
0	4							
0								
0	3							
0		19	0.93	0	0	0	0	0
2.5								
		16	14.2	2.5	1.7	9.4	0	
0	12	18	0.37	0	0	0	0	
0								
0	43							
0								
		17	3.69	3.43	0	0	0	
1								
0								
	98							
0								
0								
		15	1.82	0.39	13.7	0	0.57	0
		25	2.74	4.51	0.37	0	0	0
0	26							

11.2		15	2.96	0.76	0.55	1.3		0
0		21	0.99	0	0	0	0	
		24	47.6	0.58	0		0	0
	200							
	51							
0	482							
0	356							
0								
0	18	19	0	0	0	0	0	0

ゴマ3	そば3	えび3	いわし3	ハウスダスト3	ダニ3	総IgE3	検査時年[illegible]	卵4
sesame3	buckwheat	shrimp3	sardine3	housedust3	mite3	IgE3	agerast4	egg4
4.89		0			0	110		
0	0	0	0.35E		0			
0	0	0	0		0			
0	0	0	0				26	3.6
				4	3	503	22	82.8
0		0		2.53	3.11			
0	0	0	0		0	65		
				2	2		35	26.7
0	0	0	0		0	10		
0	0	1.26	0		1.26	75		
0	0	0	0		0		25	0.57
0	0	0	0					
0.44	0	0	0	5.79	6.27	148		
0	0	0	0	100	97		77	0
0	0	0	0		0			
0	0	0	0		0	259		

0	0	0	0				19	10.7
0.59	0	0	0		0		33	3.13
57.5	10.6		17.7				3歳8ヶ月	100
0	0	0	0		0			
	0.44			80.1	100	1113	3歳3ヶ月	25.7
1.56		0	0.36		72	1222	4歳0ヵ月	6.57
0.59	0			17.2	23.2		2歳6ヵ月	1.45
0.61	0			48.6	54.5		2歳6ヵ月	14.4
9.41	6.93	0		45	43.5		2歳6ヵ月	3.58
0	0	0	0		0			
0.35	0	0	0			140		
0	0	0	0					
0.74	0	0	0		0			
0	0	0	0	1.28	1.15			
						200	2歳5ヶ月	12.2

0	0	0	0				
1.15	0.44	0	2.09				
1.09	0.48	0	0		1.86		
0					3.47	2歳11ヶ月	20.6
0	0	0		100	100		
0	0	0	0		0		
9.87		0.48	0			1歳11ヶ月	3.14
0		0			0		
0	0	0	0			1歳11ヶ月	3.19
5.27	0.65	0	0		1.16		
0		0	0	0.67	0.83		

0	0	0	0.41		19.2	191		
0					1.81		2歳4ヶ月	0.86
				0.61	0		3歳	27
0	0	0	0			21		

牛乳4	小麦4	ピーナツ4	大豆4	米4	ゴマ4	そば4	えび4	いわし4
milk4	wheat4	peanut4	soybean4	rice4	sesame4	buckwheat	shrimp4	sardine4
0	0.38	0	0	0	0	0	0	0
0	78.6	24	5.9	1.29	14.8	2.63		0.38
8.26	35.4	16.3	13.5		15.4		2.03	9.84
0	0	0	0	0	0	0		
0	0	0	0	0	0	0	0	0

1.35	0	0	0		0			0
0.38	0	0	0		0	0	0	0
5.91	86.8	9.16	20.9	41	74.2	8.58	4.62	17.4
1.43	0.84	10.4	2.42	0.65	7.21	1.12	0	0.67
18.3	1.6	2.07	1.34	1.23	3.32		0	0
0	0.97	0	0	0	0	0		
10.9	7.03	0	0	0	0.52	0		
0	5.04	5.1	4.81	6.17	6.72	5.1	0	0
0.62	0.98	0.48	0.73	3.46	8.22	0.86	1.03	0

0	1.96	2.73						
0.65	0	4.59	0		4.1	0.52	0	0
4.22		0	0.36		0.41		0	

0	0	0	0	0	0		0	
0.59	0	0	0	0	0	0	0	0

ハウスダスト4	ダニ4	総IgE4	その他4－1	その他4－2	検査時年齢	卵5	牛乳5	小麦5
housedust4	mite4	IgE4	other41	other42	agerast5	egg5	milk5	wheat5
	0							
			鶏肉0.44		29	100	0	98.3
	100	1907	牛肉3.41		43	37	7.46	42.3
0	0							

80.6	97.1	410	

マグロ、サバ、アジ、0

0

51 100 5.74 47.4

100 100 1399

2865

18.2 20.9 112

38.7 193 ネコ1 イヌ2

39 1.71 0 2.49

31.9 イヌ48.50 37 4.18 0 0

38.3	40.6		ネコ24.4スギ8.01	43	12.9	0	0.55
		44					
	0	102		29	1.38	0.64	0

	0.6	0.67	132						
							48	13.1	0

ピーナツ5	大豆5	米5	ゴマ5	そば5	えび5	いわし5	ハウスダスト5	ダニ5
peanut5	soybean5	rice5	sesame5	buckwheat	shrimp5	sardine5	housedust5	mite5
16.7	1.74	0.77	4.77	0.87	22.5	0.6	クラス4	クラス4
24.5	17.4	4.46	15.7	8.37	3.54	11.9		

7.69	17.4	47.4	65.2		4.91	15		
2.91	2.08	3.03	3.19	2.55	0			50.5
0	0	2.23	3	0.48	0.38	0		

0.64	0	0.41	0	0	

0	0	0	171

27.5	30.1

			2.アレルギ	5.出生時の	14.母の飲酒習慣		母喫煙	
							現在	妊娠中
総IgE5	cesd	PSS		（週）	妊娠中	授乳時期	母	
IgE5	cesd	PSS	allergycon	pregnancy	drinking		smokingm	pregsmoki
			2	41	1	1	1	なし
			3	39	2	2	2	
			1	39	2	2	2	
	3	27	2	39	2	2	2	
	7	21	1	41	2	2	2	
	12	17	1	40	2	2	2	
	12	26	3	39	2	2	2	
	3	26	3	39	2	2	2	
	12	26	2	39	2	2	2	
			2	36	2	2	2	
			2	40	2	2	2	
	18	34	2	39	2	2	1	あり
	5		2	39	2	2	2	
	11	24	2	39	1	1	2	
	11	28	2	38	2	2	2	
	9	19	2	39	2	2	2	
	15	26	2	40	2	2	2	
	9	26	2	39	2	2	2	
	10	17	2	38	2	2	2	
	10	34	1	40	2	2	2	
	12	25	2	27	2	2	2	
	12	34	2	38	2	2	2	
	16	34	2	40	2	2	2	
	13	35	3	39	1	1	2	
			3	39	1	1	2	
	14	31	2	37	2	2	2	
	9	22	2	40	1	1	2	
			2	40	2	2	1	なし
			3	40	1	1	2.0	
	2	18	2	40	2	2	2.0	
	0	15	2	38	2	2	2.0	
			3		1	1	2.0	
	13	23	3	40	2	2	2.0	
	9	17	2	40	2	2	2.0	
	5	24	2	40	2	2	2.0	
			3	37	2	2	2.0	
	6	28	2	36	2	2	2.0	
	0	16	1	38	2	2	2.0	
	7	19	2	38	1	1	2.0	
	4	19	2	37	2	1	2.0	
	16	26	1	37	2	2	2.0	
	9	23	1	39	2	2	2.0	
	9	19	3	35	2	2	2.0	
	5	24		37	2	2	2.0	なし
	14	26	2	36	2	2	2.0	なし
	20	31	2	38	2	1	2.0	
	19	29	2	37	1	1	2.0	
	3	19	2	38	2	2	2.0	
	1	23	2	37	2	2	2.0	
	6	23	2	37	2	2	2.0	
			2	37	1	1	2.0	
			2		2	2	2.0	
	12	28	2	26	2	2	2.0	
	8	23	2	42	1	1	2.0	
	8	28	2	41	2	2	2.0	

	19	29	2	40	2	2	2.0		
			2	39	2	2	2.0		
	13	30	2	40	2	2	2.0		
	0	9	2	39	2	2	2.0		
	3	22	2	38	2	2	2.0		
	13	31	1	38	2	2	2.0		
	5	20	2	39	2	2	2.0		
	1	17	3	36	2	2	2.0		
	7	26	2	39	2	2	2.0		
	7	26	2	40	2	2	2.0		
			2	40	2	2	2.0		
	4	17	2	37	2	2	2.0		
	10	23	2	42	2	2	2.0		
	0	22	2	38	2	2	2.0		
	12	26	3	40	2	2	2.0		
	3	8	2	39	2	2	2.0		
	6	25	1	41	2	2	2.0		
	4	25	2	41	2	2	2.0		
			2	24	2	2	2.0		
	6	16	1	37	2	2	2.0		
			2	41	2	2	2.0		
			3	38	2	2	2.0		
			2	38	2	2	2.0		
			2	40	2	2	2.0		
	22	35	2	36	2	2		1	なし
	22	35	2	36	2	2		1	なし
	5	19	3	37	2	2	2.0		
	5	19	2	37	2	2	2.0		
			3	37	2	2	2.0		
	8	20	2	38	2	2	2.0		
	12	29	2	41	2	2	2.0		
	7	19	2	40	2	2	2.0		
			2	40	2	2	2.0		
	16	26	2	40	2	2	2.0		
	7	22	2	40	2	2	2.0		
	9	24	2	38	2	2		1	なし
195			2		2	2	2.0		
			2		2	2	2.0		
	10	22	2	38	2	2	2.0		
	29	37	3	36	2	2	2.0		
			3	41	2	2	2.0		
	11	31	2		2	2	2.0		
			3	40	2	2	2.0		
			2	40	2	2		1	なし
			3	39	2	2	2.0		
	0	12	2	39	2	2	2.0		
	10	24	2	37	2	2	2.0		
	8	26	2	40	2	2	2.0		
	5	26	2	40	2	2	2.0		
			2	37	2	2	2.0		
	8	20	2	41	2	2	2.0		
			2	38	2	2	2.0		
	1	20	2	39	2	2	2.0		
	14	31	2	38	2	2	2.0		
	10	39	2	32	2	2	2.0		
	19	29	3	39	2	2	2.0		
	22	29	3	39	2	2	2.0		
			2		2	2	2.0		
	20	30	2	39	2	2	2.0		

	29	3	38	2	2	2.0		
1	24	2	38	2	2	2.0		
11	21	1	40	2	2		1	あり
21	36	3	39	2	2	2.0		
10	26	2	39	2	2	2.0		
8	28	3	38	2	2	2.0		
8	19	2	42	2	2	2.0		
		1	39	2	2	2.0		
3	25	3	39	2	2	2.0		
16	42	2	40	2	2	2.0		
12	35	2	38	2	2	2.0		
8	23	3	39	2	2	2.0		
7	15	2	40	2	2	2.0		
7	26	2	38	2	2	2.0		
11	23	2	40	2	2	2.0		
12	27	2	40	2	2	2.0		
9	27	2	40	2	2	2.0		
		3	41	2	2	2.0		
		2	40	2	2	2.0		
34	26	3	38	2	2	2.0		
6	16	2	37	2	2	2.0		
		2	40	2	2		1	
3	13	3	37	2	2	2.0		
8	32	3	42	2	2	2.0		
0	3	2	40	2	2	2.0		
5	16	2	40	2	2	2.0		
	19	2	39	2	2	2.0		
15	21	2	40	2	2	2.0		
1	12	2	36	2	2	2.0		
9	26	1	38	2	2	2.0		
1	12	1	39	2	2	2.0		
9	23	3	40	2	2	2.0		
8	23	2	41	2	2	2.0		
20	27	2	38	2	2	2.0		
1	10	1	37	2	2	2.0		
9	35	3	40	2	2	2.0		
0	10	1	40	2	2	2.0		
0	7	2	39	2	2	2.0		
5	22	2	36	2	2	2.0		
6	18	3	40	2	2	2.0		
1	14	3	29	2	2	2.0		
1	11	2	39	2	2	2.0		
0	10	3	40	2	2	2.0		
0	17	2	38	2	2	2.0		
22	37	2	38	2	2	2.0		
14	23	2	39	2	2	2.0		
0	9	1	38	2	2	2.0		
2	15	2	38	2	2	2.0		
4	21	2	40	2	2	2.0		
10	25	2	39	2	2	2.0		
		1	40	2	2	2.0		
9	25	3	39	2	2	2.0		
5	8	2	39	2	2	2.0		
1	10	2	40	2	2	2.0		
3	20	2	38	2	2	2.0		
12	26	2	40	2	2	2.0		
12	26	3	37	2	2	2.0		
3	17	3	42	2	2		1	あり
		2	40	2	2	2.0		

			2	40	2	2	2.0
			2	40	2	2	2.0
	1	10	1	38	2	2	2.0
	0	7	2	40	2	2	2.0
			2	39	2	2	2.0
	1	13	2	38	2	2	2.0
	2	19	2	39	2	2	2.0
	1	12	2	38	2	2	2.0
485	10	30	2	39	2	2	2.0
			2	40	2	2	2.0
			2	39	2	2	2.0
	6	21	2	41	2	2	2.0
	1	10	2	39	2	2	2.0
	6	30	2	39	2	2	2.0
	3	11	2	38	2	2	2.0
	6	20	2	39	2	2	2.0
			3	40	2	2	2.0
	7	23		41	2	2	2.0
	7	19	2	37	2	2	2.0
	15	27	2	39	2	2	2.0
	0	15		38	2	2	2.0
	21	30	3	38	2	2	2.0
	12	24	2	37	2	2	2.0
	12	24	2	37	2	2	2.0
	2	11	3	39	2	2	2.0
	2	14	1	39	2	2	2.0
	14	36	2	38	2	2	2.0
	6	16	2	40	2	2	2.0
	0	10	2	38	2	2	2.0

	agemother		
smoking	agemother	dustclass	dustno
1	26	0	0
1	34	0	0
2	30	0	0
1	41	0	0
1	36	0	0
2	30	0	0
2		0	0
2		0	0
2	24	4	33.75
2	31	2	3.11
1		0	0
1	32	0	0
2	32	0	0
2	33	0	0
1	29	0	0
2	33	6	100
1	37	0	0
1	33	0	0
2	33	0	0
1	43	0	0
1	28	2	1.26
1		0	0
1	23	0	0
2	33	0	0
2		5	68.8
1		3	3.83
2	33	0	0
1	34	2	0.99
2		0	0
1	30	0	0
2	37	0	0
2	32	0	0
2		0	0
2	37	0	0
1	34	0	0
1	33	6	100
1	40	0	0
1	26	3	6.44
1	22	0	0
2	22	0	0
1	29	0	0
2	28	0	0
1	29	0	0
2	29	3	6.27
1	33	0	0
1	28	0	0
1	33	0	0
2		5	97.1
1		0	0
1	31	0	0
2	37	0	0
1	28	0	0
2	30	0	0
2		0	0
2	30	0	0

2	30	0	0
2	30	0	0
1	43	0	0
1	35	0	0
1	36	0	0
1		0	0
1		0	0
2		1	0.59
2	30	3	13.3
2	41	0	0
2	34	5	52.4
2	34	0	0
1	29	0	0
2	35	0	0
1	32	1	0.48
2		1	0.6
2	36	0	0
2		0	0
2			
1		0	0
1		0	0
2	30	6	100
2	30		
2	36	5	72
1	36	4	21.7
1		0	0
1	31	4	20.9
1	39	4	38.7
1	30	5	57.8
1		0	0
1	30	0	0
1	31	0	0
1	26	0	0
1		0	0
1		0	0
1	32	0	0
2	33	5	50.5
2		0	0
1	30	0	0
2		2	2.61
1		0	0
2		0	0
1	33		
1	30	4	33.1
2	35	0	0
2	31	0	0
1		0	0
2	31		
2		0	0
2	32	0	0
1	36	0	0
1	32	0	0
1	29	1	0.99
2	29	0	0
1		0	0
1	40	4	31.9
1		0	0
1	26	0	0
1	26		

1	30		
1	29	0	0
1	39		
2	31	2	1.86
2		0	0
1	32	0	0
2	33	0	0
2	30	0	0
2	31	0	0
1	35	2	0.88
2	26	2	1.25
2	29	0	0
2	25	0	0
2	28	6	100
1		0	0
1		0	0
1	30	0	0
2	22	0	0
2		0	0
1	30	0	0
1	35	0	0
1	33	0	0
2	30	0	0
2		0	0
2	30	0	0
1	30	0	0
1	30	0	0
2	30	0	0
2	27	0	0
2	32	0	0
2	33	0	0
2	30	4	17.7
2	34	0	0
1	25	5	10.3
2	30	0	0
2	30	2	1.71
1	37	0	0
2	25	0	0
1	34	0	0
2	27	6	100
1	35	0	0
1	25	0	0
1	35	0	0
1	35	0	0
1	28	0	0
1	30	0	0
2	32	0	0
1		0	0
2	35		
2	43	4	33.7
2	25		
2	29		
1	30	0	0
2	37		
1	35	0	0
1		0	0
1		0	0
1		0	0
2	30		

2	25		
2			
1	32	0	0
1	30	4	19.2
1	30		
2	30	0	0
2		0	0
1		0	0
2	32	4	30.1
2	30		
1	28		
2	28	3	17.1
2	27	0	0
2		0	0
1	25	0	0
2	28	0	0
1	25	0	0
1	25	5	33.2
1	31	0	0
1	35	0	0
1	35	0	0
2	33	0	0
1	25	0	0
1	22	0	0
2	25	0	0
1	25	0	0
1	30	0	0
1		0	0
2		0	0

4
